# Comparison of different detection methods for *Mycoplasma pneumoniae* infection in children with community-acquired pneumonia

**DOI:** 10.1186/s12887-021-02523-4

**Published:** 2021-02-19

**Authors:** Mingyu Tang, Dong Wang, Xing Tong, Yufen Wu, Jing Zhang, Lei Zhang, Yong Yin, Qing Cao

**Affiliations:** 1 Department of Respiratory Medicine, Shanghai Children’s Medical Center, Shanghai Jiao Tong University School of Medicine, 1678 Dongfang Rd, Shanghai, 200127 China; 2 Department of Infectious Diseases, Shanghai Children’s Medical Center, Shanghai Jiao Tong University School of Medicine, Shanghai, 200127 China

**Keywords:** Detection methods, *Mycoplasma pneumoniae*, Children, Community-acquired pneumonia

## Abstract

**Background:**

Due to the lack of a sensitive, specific and rapid detection method, aetiological diagnosis of pneumonia caused by *Mycoplasma pneumoniae* (*M. pneumoniae, MP*) is a constantly challenging issue. This retrospective study aimed to compare the diagnostic methods for *Mycoplasma pneumoniae* in children and evaluate their values.

**Methods:**

From November 2018 to June 2019, 830 children with community-acquired pneumonia were selected from the Department of Respiratory Medicine, Shanghai Children’s Medical Center. On the first day of hospitalization, sputum, throat swab and venous blood samples were collected to analyse MP-IgM (particle agglutination, PA), MP-IgM (immune colloidal gold technique, GICT), MP-DNA, MP-RNA (simultaneous amplification and testing, SAT) and MP-DNA (real-time polymerase chain reaction, RT-PCR).

**Results:**

Among these 830 children, RT-PCR showed that the positive rate was 36.6% (304/830), in which the positive rate of macrolide resistance (A2063G mutation) accounted for 86.2% of cases (262/304). Using RT-PCR as the standard, MP-RNA (SAT) had the highest specificity (97.5%), and MP-IgM (PA) had the highest sensitivity (74.0%) and Youden index (53.7%). If MP-RNA (SAT) was combined with MP-IgM (PA), its Kappa value (0.602), sensitivity (84.2%), specificity (78.7%) and Youden index (62.9%) were higher than those of single *M. pneumoniae* detection.

**Conclusions:**

Our research indicated that a combination of MP-RNA (SAT) plus MP-IgM (PA) might lead to reliable results as an early diagnostic method for children with clinical manifestations of *Mycoplasma pneumoniae* pneumonia.

**Supplementary Information:**

The online version contains supplementary material available at 10.1186/s12887-021-02523-4.

## Background

*Mycoplasma pneumoniae* (*M. pneumoniae, MP*) is known as a common cause of community-acquired pneumonia (CAP) in children throughout the world. *M. pneumoniae* is the smallest prokaryotic microorganism, lacks a cell wall and has a high affinity for respiratory epithelial cells. A total of 40% or more cases of CAP in children are caused by *M. pneumoniae,* and approximately 18% of cases require hospitalization [1]. At present, it is believed that the pathogenic mechanism of *M. pneumoniae* is to cause direct injury through community-acquired respiratory distress syndrome (CARDS) toxin, which induces eosinophilia, increases cytokine production and induces the hyperreactivity of the airway, such as asthma, in animal models [2–4]. In addition, the immunological response resulting from infection by *M. pneumoniae* causes pulmonary and extrapulmonary symptoms [5].

A worldwide increase in the prevalence of macrolide-resistant *mycoplasma pneumoniae* (MRMP) strains has been witnessed since 2000 [6]. In some regions of Asia, the resistance rates have been reported to be over 90% [7], whereas in North America and Europe, the rates have reached 25% [8, 9]. Compared to other patients with CAP, children with *M. pneumoniae* infection may be older and have prolonged symptoms of fever and cough [10]. The period of signs and symptoms will be shortened if accurate antimicrobial treatment is started early in the course of diseases. However, *Mycoplasma pneumoniae* pneumonia (MPP) in children cannot be diagnosed exactly in terms of clinical manifestations [11].

*Mycoplasma pneumoniae* pneumonia has the characteristics of insidious onset, mild pulmonary signs and low specificity of clinical manifestations and imaging. Therefore, effective and sensitive laboratory diagnostic methods are the main basis for the diagnosis of *M. pneumoniae* infection. At present, there are many clinical methods for detecting *M. pneumoniae*, such as culture, serology and molecular-based methods. However, as the gold standard, culture is limited in clinical utility because it is time-consuming and insensitive [12]. Both serology and molecular assays have their own advantages and disadvantages in clinical practice. In this paper, we aimed to evaluate the clinical application value of different methods to detect *M. pneumoniae* infection by comparing and analysing them to improve diagnostic efficiency.

## Methods

### Study population

We conducted a retrospective study that included children aged 1 month to 15 years with radiologically confirmed community-acquired pneumonia. Venous blood, throat swab and sputum specimens were obtained from these patients on the day of hospitalization at the Department of Respiratory Medicine, Shanghai Children’s Medical Center (SCMC) from November 1, 2018, to June 30, 2019. These specimens were tested for MP-IgM (particle agglutination, PA), MP-IgM (immune colloidal gold technique, GICT), MP-RNA (simultaneous amplification and testing, SAT), 7 respiratory tract RNAs (7 RNAs) and MP-DNA (real-time polymerase chain reaction, RT-PCR).

### MP-IgM (particle agglutination, PA)

Particle agglutination (PA) antibody titres for *M. pneumonia* were assayed using SERODIA MYCO-II (Fuji Rebio Ltd., Tokyo, Japanese), which was performed using artificial gelatine particles sensitized with cell membrane components of *M. pneumoniae*. The result was considered positive if the titre was 1:160 or more (≥1:160).

### MP-IgM (immune colloidal gold technique, GICT)

According to the manufacturer’s (Alere Diagnostics, Shanghai, China) instructions, colloidal gold was used as a tracer marker to detect *M. pneumoniae* antibodies using the principle of immunoreaction of specific antigen antibodies. The results of this method showed only negative, weak positive or positive titres.

### MP-RNA (simultaneous amplification and testing, SAT)

Throat swab specimens of the children were collected and tested using *M. pneumoniae* nucleic acid detection kits (Rendu Biotechnology, Shanghai, China), including nucleic acid extraction and isothermal amplification detection.

### Seven respiratory tract RNAs (7 RNAs)

Based on the double-amplification method of RNA isothermal amplification and multiple biotin signals, throat swab samples were collected to identify seven common respiratory pathogen RNAs (Zhongzhi Biotechnologies, Wuhan, China), namely influenza A, influenza B, respiratory syncytial virus (RSV), parainfluenza virus, adenovirus, *Mycoplasma pneumoniae* and *Chlamydia pneumoniae*, in a short period of time.

### MP-DNA (real-time polymerase chain reaction, RT-PCR)

According to the specific point mutations in domain V of MP 23S rRNA (at positions 2063 and 2064), the primers were designed as follows: forward primer: 5′-AACTATAACGGTCCTAAGGTAGCG-3′; reverse primer: 5′-GCTCCTACCTATTCTCTACATGAT-3′. Sputum aspirated from the patients was collected for sample processing, and DNA was extracted using a QIAamp DNA Mini Kit (Qiagen, Hilden, Germany). PCR was performed, and the PCR product was verified using gel electrophoresis. Finally, the positive specimen was sequenced and then analysed using SnapGene software (from Insightful Science; available at snapgene.com). The measured sequence was compared to the standard sequence of the M129 strain in the NCBI database. If the sequence was not consistent, the A2063G transition was considered to be involved in macrolide resistance. Unfortunately, we did not find a mutation at position 2064.

### Statistical analysis

SPSS software package v21.0 was used for all statistical analyses. Categorical variables are expressed as frequencies and percentages. Four-table diagnostic tests were used to calculate the Kappa value, sensitivity, specificity and Youden index. In a previous report [13], the value of the Kappa statistic was interpreted as follows: < 0.01, poor; 0 to 0.20, slight; 0.21 to 0.4, fair; 0.41 to 0.6, moderate; 0.61 to 0.80, substantial; and 0.81 to 1, almost perfect. The other indices were calculated using the following formulas: sensitivity = true positive cases / (true positive cases + false negative cases) × 100%; specificity = true negative cases / (true negative cases + false positive cases) × 100%; and Youden index = sensitivity + specificity − 1. The Youden index was used to evaluate the effectiveness and authenticity of experiments.

## Results

### Clinical characteristics of patients

A total of 830 patients diagnosed with community-acquired pneumonia, aged 1 month to 15 years, were enrolled in the present study between November 1, 2018, and June 30, 2019. Congenital heart disease, congenital biliary atresia, epilepsy, hydrocephalus and cerebral palsy were the most frequently observed underlying diseases in these patients (Table 1).

**Table 1:** General characteristics of the patients

Characteristic	Value for patients
Total	830
Age, No. (%)	
< 3 years	421 (50.7)
3–5 years	215 (25.9)
6–15 years	194 (23.4)
Sex, No. (%)	
Male	447 (53.9)
Female	383 (46.1)
Underlying diseases, No. (%)	
None	680 (81.9)
Congenital heart disease	101 (12.2)
Congenital biliary atresia	7 (0.8)
Brain lesions	15 (1.8)
Other diseases	27 (3.3)
Need of oxygen	
Yes	61 (7.3)
No	769 (92.7)
Prognosis after treat, No. (%)	
Alive	825 (99.4)
Death^a^	5 (0.6)

^a^Four children died from congenital heart disease, and one died from primary immunodeficiency disease

### Comparison of positive rates of *M. pneumoniae* detection methods in different age groups

All the patients were grouped by age as follows: infants (age: < 3 years), preschoolers (age: 3–5 years) and school-aged children (age: 6–15 years). The highest specimen positivity rate was in school-aged children. There were significant differences in the positivity rate of the different age groups by each detection method, except for the 7 RNAs method (Table 2). In addition, the rate of macrolide resistance was highest in school-aged children (87.9%, 123/140) and lowest in infants (82.3%, 65/79), but it was not statistically significant (X^2^ = 1.396, *P* < 0.001).

**Table 2:** Positive rates of methods in different age groups

Methods	< 3 years	3-5 years	6-15 years	X^2^	p
	*n* = 421(%)	*n* = 215(%)	*n* = 194(%)		
MP-IgM (PA)	91 (21.6)	102 (47.4)	139 (71.6)	145.222	< 0.001
MP-IgM (GICT)	66 (15.7)	67 (31.2)	89 (45.9)	64.704	< 0.001
MP-RNA (SAT)	36 (8.6)	40 (18.6)	70 (36.1)	69.646	< 0.001
7RNAs	71 (16.9)	29 (13.5)	38 (19.6)	2.772	0.25
RT-PCR	79 (18.8)	85 (39.5)	140 (72.2)	164.208	< 0.001
Resistance	65 (15.4)	74 (34.4)	123 (63.4)	142.516	< 0.001

*SAT* simultaneous amplification and testing

*PA* particle agglutination

*GICT* immune colloidal gold technique

*RT-PCR* real-time polymerase chain reaction

### Positive rates of different detection methods for *M. pneumoniae*

**Fig. 1:**
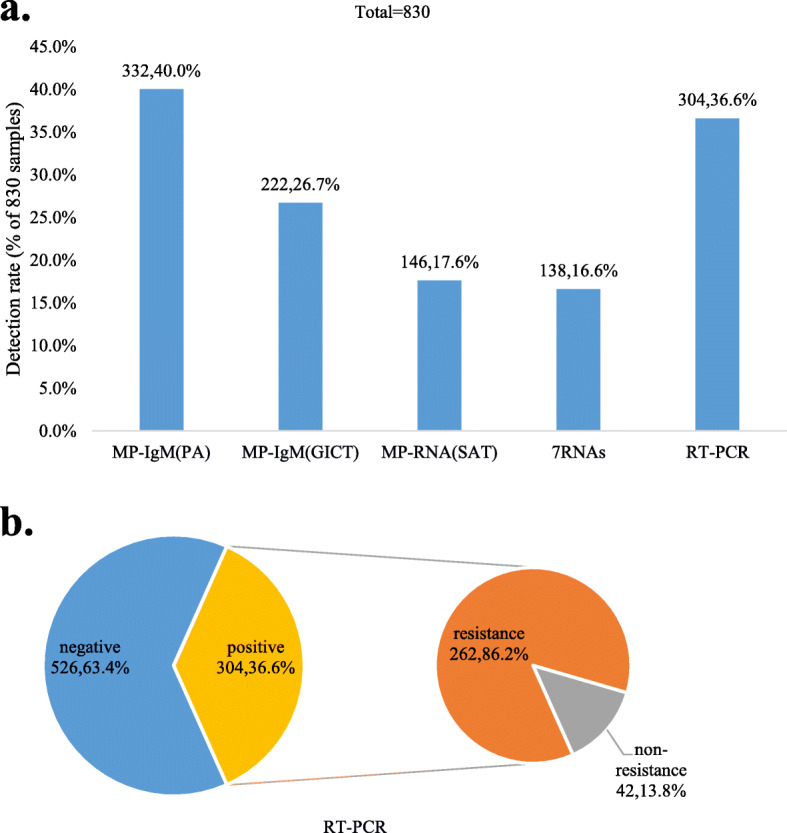
Positive rates of different detection methods for *M. pneumoniae*. A. The positive rate of MP-IgM (PA) was higher than that of the other methods. B. The positive rate of RT-PCR for *M. pneumoniae* was 36.6%, and the rate of macrolide resistance accounted for 86.2% of cases by sequencing analysis

In these 830 children, the positive rates of MP-IgM (PA), MP-IgM (GICT), MP-RNA (SAT) and 7 RNAs were 40% (332/830), 26.7% (222/830), 17.6% (146/830) and 16.6% (138/830), respectively. MP-DNA (RT-PCR) results showed that the positive rate was 36.6% (304/830), of which macrolide resistance accounted for 86.2% of cases (262/304) (Fig. 1). Through 7 RNAs, we found that the coinfection combination containing MP and RSV had the highest detection rate of 23.9% (33/138) followed by MP and parainfluenza virus (21.7%, 30/138).

### Diagnostic value of different methods with a real-time polymerase chain reaction as the standard

We compared the diagnostic values of MP-IgM (PA), MP-IgM (GICT), MP-RNA (SAT) and 7 RNAs, considering MP-DNA (RT-PCR) as the standard. As shown in Table 3, MP-IgM (PA) had a good consistency (Kappa = 0.526), sensitivity (74.0%) and Youden index (53.7%). The specificity of MP-RNA (SAT) was the highest (97.5%) followed by 7 RNAs (89.2%). Moreover, comparing every two detection methods together, we found that the combination of MP-IgM (PA) and MP-RNA (SAT) was the best choice to detect *M. pneumoniae* because its substantial Kappa value (Kappa = 0.602) and its highest sensitivity (84.2%), specificity (78.7%) and Youden index (62.9%) were greater than those of the single method (Table 4).

**Table 3:** Diagnostic values of different methods with a real-time polymerase chain reaction as the standard

	RT-PCR		Kappa	Sensitivity	Specificity	Accuracy	Youden Index
	Positive	Negative					
**MP-IgM (PA)**							
Positive	225	107	0.526	74.0%	79.7%	77.6%	53.7%
Negative	79	419					
**MP-IgM (GICT)**							
Positive	151	71	0.384	49.7%	86.5%	73.0%	36.2%
Negative	153	455					
**MP-RNA (SAT)**							
Positive	133	13	0.464	43.8%	97.5%	77.8%	41.2%
Negative	171	513					
**7 RNAs**							
Positive	81	57	0.179	26.6%	89.2%	66.3%	15.8%
Negative	223	469					

*SAT* simultaneous amplification and testing

*PA* particle agglutination

*GICT* immune colloidal gold technique

**Table 4:** Diagnostic value of combining different methods with a real-time polymerase chain reaction as the standard

	RT-PCR		Kappa	Sensitivity	Specificity	Accuracy	Youden Index
	Positive	Negative					
**MP-RNA (SAT) + MP-IgM (PA)**							
Positive	256	112	0.602	84.2%	78.7%	80.7%	62.9%
Negative	48	414					
**MP-RNA (SAT) + MP-IgM (GICT)**							
Positive	223	76	0.591	73.4%	85.6%	81.1%	59.0%
Negative	81	450					
**MP-IgM (GICT) + MP-IgM (PA)**							
Positive	237	123	0.525	78.0%	76.6%	77.2%	54.6%
Negative	67	403					
**7 RNAs + MP-IgM (PA)**							
Positive	250	154	0.419	82.2%	70.7%	74.9%	52.9%
Negative	54	372					
**7 RNAs + MP-IgM (GICT)**							
Positive	198	120	0.419	65.1%	77.2%	72.8%	42.3%
Negative	106	406					

*SAT* simultaneous amplification and testing

*PA* particle agglutination

*GICT* immune colloidal gold technique

## Discussion

In this study, we assessed the detection rate of different methods for *M. pneumoniae* pneumonia in different age groups and compared the value of these diagnostic methods.

*M. pneumoniae* is a major cause of infectious diseases worldwide. *M. pneumoniae* affects different tissues and organs, especially the respiratory tracts, of children in all age groups, and it has become the second leading pathogen after *Streptococcus pneumoniae* in children with community-acquired pneumonia [14]. The occurrence of CAP caused by *M. pneumoniae* varies with age. A previous study has reported that *M. pneumoniae* pneumonia is uncommon in children under 5 years of age but has a higher incidence among school-age children [1]. However, *M. pneumoniae* infections may occur in people from infancy through old age [15, 16]. From 2010 to 2012, 2638 children with pneumonia requiring hospitalization in three hospitals in the United States were enrolled in a study. Respiratory specimens were systematically collected and detected using real-time PCR, and *M. pneumoniae* was found to be more common in children ≥5 years (19% vs. 3%) [17]. In accordance with our data, the detection rates of *M. pneumoniae* by different methods in the three age groups were different, but the distributions were the same. School-aged children and adolescents were the most common ages affected, and there was rarely a significant infection before 3 years old, which was consistent with the epidemiological characteristics of *M. pneumoniae*.

At present, there are many methods for the laboratory diagnosis of *M. pneumoniae* infection, including culture, serological detection and molecular assays. Culture is often used for antimicrobial susceptibility testing or typing due to its high specificity. However, due to the complicated procedure, prolonged turnaround time and low sensitivity, culture is not recommended for routine testing [18, 19]. Serological tests are currently the most widely used detection methods of *M. pneumoniae* in clinical practice and are more sensitive than culture. Diagnostic sensitivity for serological tests of *M. pneumoniae* is determined by both the collection time of specimens and the performance characteristics of the methods. In our study, the positive rate of MP-IgM (PA) was 40%, which was higher than that of the others, which may have been attributed to the average time being 10 days before admission to our department. Although MP-IgM is the earliest antibody produced after *M. pneumoniae* infection, it still takes a certain period of time before it can be detected. In general, MP-IgM can be detected within approximately 1 week after infection, reaching a peak after 3 to 4 weeks, resulting in possible false negatives in the early stage [20]. It has been reported that the most accurate diagnosis of serology is obtained when paired sera collected at least 2 weeks apart are tested for both IgM and IgG at the same time, resulting in a 4-fold increase in titre [1]. However, in paediatrics, it is simply impossible to perform repeated blood sampling in a short time. MP-IgM (GICT), a rapid test kit based on immunochromatography, is a relatively new serological test. One study from Wei Li found that the specificity and sensitivity of MP-IgM (GICT) were 100 and 97.4%, respectively, compared with real-time PCR [21], which was not in line with our results. It might be that the dilution of the sample to only 100 μl caused false negatives. Due to the easy and rapid procedure (15 min), MP-IgM (GICT) is suitable for the identification of *M. pneumoniae* infections in paediatric outpatient departments. However, MP-IgM (PA) is still recommended for inpatients to reduce the false-negative rate.

With the development of molecular assays, nucleic acid amplification technology (NAAT) has gradually become an important method for the early rapid diagnosis of *M. pneumoniae* infection, which may help with early appropriate antibiotic therapy. Owing to the advantages of quick turnaround times, lower likelihood of contamination, higher sensitivity and higher specificity, as well as not being limited by time and immune function, NAAT is important in the early diagnosis of *M. pneumoniae*. In addition, NAAT has various detection formats, which can provide quantitative data, detect antimicrobial resistance genes and analyse the genetic relatedness of organisms [12]. However, there are a several limitations, such as contamination, which may result in false positives; difficulty in obtaining high-quality samples; sampling time point affecting results; and the possibility of PCR inhibitors leading to false negatives [22]. These limitations may have contributed to the lower positive rate of NAAT in this study.

We found that the rate of macrolide resistance accounted for 86.2% of cases. MRMP strains have been increasing in many countries since the first case was reported in Japan in 2000 [23] with the long-term widespread use of macrolides. Macrolides act on the ribosomal 50S subunit to inhibit protein synthesis. Mutations in the V region of the MP 23S rRNA domain can cause a decrease in affinity between the drug and the ribosome, leading to drug resistance [24]. Common mutations, at both position 2063 and position 2064, lead to high-level resistance, whereas positions 2067 and 2617 are associated with low-level resistance [25, 26]. In these 262 specimens, we found a mutation only at position 2063. MRMP in the Beijing population reached a high rate of more than 90% from 2008 to 2012 [27], which was similar to our results.

MP-DNA detection by RT-PCR technology amplifies gene fragments to diagnose pathogens, which has advantages in operation and sensitivity of detection [28]. We compared the diagnostic values of different methods for *M. pneumoniae* with RT-PCR as the standard. We found that MP-IgM (PA) had a high sensitivity of 74.0% and the highest Youden index and Kappa value, indicating that it was conducive to screening for MP infection. SAT is a recently developed method based on isothermal amplification of RNA [29, 30], which can be completed in approximately 3 h. In our study, the outcomes of SAT for *M. pneumoniae*, both MP-DNA and 7 RNAs, showed higher specificity but lower sensitivity. Specific RNA exists only in the proliferation stage of *M. pneumoniae*, indicating that increases in RNA levels may reflect bacterial multiplication [31]. Previous data have shown that the SAT positivity rates are significantly higher in untreated cases with MPP than in macrolide-treated MPP cases [32]. Therefore, one explanation for the “false-negative” SAT results is that long-term treatment might decrease the *M. pneumoniae* load. Our data showed that 78.3% of the patients received macrolide treatment before hospitalization. Another explanation might relate to the possible poor sampling procedural skills and the quality of the swab samples, leading some samples to be below the assay’s detection limit [33, 34]. In addition, multiplex PCR assays can be used to detect *M. pneumoniae* and other respiratory pathogens, but monoplex assays have higher sensitivity and specificity than multiplex assays [35], which was confirmed by our results.

Each detection method of *M. pneumoniae* has its own advantages and disadvantages. Although molecular assays are superior in detecting *M. pneumoniae* due to their rapid, sensitive and specific characteristics, they cannot take the place of serology [36]. A study in China analysed data from children hospitalized with MPP using IgM (PA) and RT-PCR [37]. The concordance was close to 90% for the two detection methods. However, 173 (7%) children with a positive PCR had a negative serological test, and there were only 72 (3%) IgM-positive children who were PCR negative. Different detection methods can be used not only to improve diagnostic specificity and sensitivity but also to reduce the false-negative rate and false-positive rate. No single test available can be reliable for the identification of *M. pneumoniae* infection, and a combination of various methods is the most reliable approach [38]. A positive PCR without serological evidence of infection may indicate that the specimen was collected too early in the course of *M. pneumoniae* infection for antibodies to develop. PCR results may become negative after a period of antibiotic treatment, while serological results would remain positive for a long time. Some reviews have concluded that no single test could reliably detect *M. pneumoniae* infection, but a combination of serological tests and PCR might be the most sensitive approach for early diagnosis in children [12, 39]. In our research, two different *M. pneumoniae* detection methods were combined and evaluated by RT-PCR, and the results showed that MP-IgM (PA) in combination with MP-RNA (SAT) can be used as a good screening method.

Our study had some limitations. First, this study was performed in a single centre and included no data on clinical features because there were no accurate ways to determine the specificity of these characteristics. Second, the lack of a gold standard diagnostic assay, such as microbiological culture and a 4-fold increase in IgM and IgG in paired serum, made it difficult to draw conclusions to confirm the most reliable and accurate method.

## Conclusions

In conclusion, during the acute phases of *M. pneumoniae* pneumonia in children, the detection of MP-IgM (PA) together with MP-RNA (SAT) proved to be a reliable and precise diagnosis. Paediatricians need to select appropriate detection methods based on the age of children and the onset of the disease, and they need to consider various results to diagnose *M. pneumoniae* infection.

## Supplementary Information

**Additional file 1.**

## Data Availability

The datasets generated and/or analysed during the current study are available in the Supplementary Material.

## Abbreviations

MP: *Mycoplasma pneumoniae*
CAP: community-acquired pneumonia
PA: particle agglutination
GICT: immune colloidal gold technique
SAT: simultaneous amplification and testing
RT-PCR: real-time polymerase chain reaction
MRMP: macrolide-resistant *mycoplasma pneumoniae*
RSV: respiratory syncytial virus
