# Supplementary material for: Comparison of different detection methods for Mycoplasma pneumoniae infection in children with community-acquired pneumonia
Source: BMC Pediatr. 2021 Feb 19;21:90. doi: 10.1186/s12887-021-02523-4 (PMC7893926; doi:10.1186/s12887-021-02523-4)
Supplement: Supplementary file 1 — Additional file 1. [file 12887_2021_2523_MOESM1_ESM.docx]

**Supplementary Material：The datasets generated and/or analysed during the current study**

|  | Underlying diseases | Need of oxygen | Prognosis after treat | MP-IgM(GICT) | 7RNAs | MP-RNA | MP-IgM(PA) | MP-PCR | Resistance |
| --- | --- | --- | --- | --- | --- | --- | --- | --- | --- |
| 1 | Congenital heart disease | No | Alive | Negative | Negative | Negative | Negative | Negative |  |
| 2 | None | No | Alive | Negative | Negative | Negative | 1:40 | Negative |  |
| 3 | Brain lesions | No | Alive | Negative | Negative | Negative | Negative | Negative |  |
| 4 | None | No | Alive | Positive | Negative | Negative | 1:160 | Negative |  |
| 5 | None | No | Alive | Positive | Negative | Negative | 1:160 | Negative |  |
| 6 | None | No | Alive | Negative | Negative | Negative | Negative | Negative |  |
| 7 | None | No | Alive | Positive | Negative | Negative | 1:160 | Negative |  |
| 8 | None | No | Alive | Positive | Negative | Negative | 1:160 | Negative |  |
| 9 | None | No | Alive | Positive | Negative | Positive | 1:160 | Negative |  |
| 10 | None | No | Alive | Negative | Negative | Negative | 1:80 | Negative |  |
| 11 | None | No | Alive | Negative | Negative | Negative | 1:160 | Negative |  |
| 12 | None | No | Alive | Negative | Positive | Negative | 1:160 | Negative |  |
| 13 | None | No | Alive | Negative | Negative | Negative | 1:160 | Negative |  |
| 14 | None | No | Alive | Negative | Negative | Negative | 1:160 | Negative |  |
| 15 | None | No | Alive | Negative | Positive | Negative | Negative | Negative |  |
| 16 | Congenital heart disease | No | Alive | Negative | Negative | Negative | 1:160 | Negative |  |
| 17 | None | No | Alive | Negative | Negative | Negative | Negative | Negative |  |
| 18 | None | No | Alive | Positive | Negative | Positive | 1:160 | Negative |  |
| 19 | None | No | Alive | Positive | Negative | Negative | 1:160 | Negative |  |
| 20 | None | No | Alive | Negative | Negative | Positive | 1:80 | Positive | Yes |
| 21 | None | No | Alive | Negative | Negative | Negative | Negative | Negative |  |
| 22 | Congenital heart disease | No | Alive | Negative | Negative | Negative | 1:80 | Negative |  |
| 23 | Congenital heart disease | No | Alive | Negative | Negative | Negative | Negative | Negative |  |
| 24 | None | No | Alive | Negative | Negative | Negative | 1:80 | Negative |  |
| 25 | None | No | Alive | Negative | Negative | Negative | 1:160 | Negative |  |
| 26 | None | No | Alive | Positive | Negative | Negative | 1:160 | Positive | Yes |
| 27 | None | No | Alive | Positive | Negative | Negative | 1:160 | Negative |  |
| 28 | None | No | Alive | Positive | Negative | Positive | 1:160 | Positive | Yes |
| 29 | None | No | Alive | Negative | Negative | Negative | 1:40 | Negative |  |
| 30 | None | No | Alive | Negative | Negative | Negative | 1:160 | Negative |  |
| 31 | None | No | Alive | Negative | Negative | Negative | Negative | Negative |  |
| 32 | None | No | Alive | Negative | Positive | Negative | 1:80 | Negative |  |
| 33 | None | No | Alive | Negative | Negative | Negative | 1:40 | Negative |  |
| 34 | Congenital biliary atresia | No | Alive | Negative | Negative | Negative | 1:40 | Negative |  |
| 35 | None | No | Alive | Negative | Negative | Negative | 1:160 | Negative |  |
| 36 | None | No | Alive | Positive | Negative | Negative | 1:160 | Positive | Yes |
| 37 | None | No | Alive | Positive | Negative | Negative | 1:160 | Positive | Yes |
| 38 | None | No | Alive | Negative | Negative | Negative | 1:40 | Negative |  |
| 39 | None | No | Alive | Negative | Negative | Negative | Negative | Negative |  |
| 40 | None | No | Alive | Positive | Positive | Positive | 1:160 | Negative |  |
| 41 | Congenital anal atresia | No | Alive | Negative | Negative | Negative | Negative | Negative |  |
| 42 | None | No | Alive | Negative | Negative | Negative | Negative | Negative |  |
| 43 | None | No | Alive | Negative | Negative | Negative | Negative | Negative |  |
| 44 | None | No | Alive | Negative | Negative | Negative | Negative | Negative |  |
| 45 | None | No | Alive | Positive | Negative | Negative | 1:160 | Positive | Yes |
| 46 | None | No | Alive | Positive | Negative | Positive | 1:80 | Positive | Yes |
| 47 | None | No | Alive | Negative | Negative | Negative | Negative | Negative |  |
| 48 | None | No | Alive | Negative | Negative | Negative | Negative | Positive | Yes |
| 49 | None | No | Alive | Positive | Negative | Negative | 1:160 | Negative |  |
| 50 | None | No | Alive | Negative | Positive | Negative | Negative | Positive | Yes |
| 51 | None | No | Alive | Positive | Positive | Negative | 1:160 | Positive | Yes |
| 52 | None | Yes | Alive | Negative | Positive | Negative | Negative | Positive | No |
| 53 | None | No | Alive | Positive | Negative | Negative | 1:40 | Negative |  |
| 54 | None | No | Alive | Negative | Negative | Negative | 1:160 | Positive | Yes |
| 55 | None | No | Alive | Negative | Negative | Positive | 1:80 | Positive | Yes |
| 56 | None | No | Alive | Positive | Negative | Negative | Negative | Negative |  |
| 57 | None | No | Alive | Negative | Negative | Negative | 1:40 | Negative |  |
| 58 | None | No | Alive | Negative | Negative | Negative | Negative | Negative |  |
| 59 | None | No | Alive | Negative | Negative | Negative | 1:160 | Negative |  |
| 60 | None | No | Alive | Positive | Negative | Positive | 1:160 | Positive | Yes |
| 61 | None | Yes | Alive | Negative | Negative | Negative | Negative | Negative |  |
| 62 | None | No | Alive | Negative | Negative | Negative | Negative | Negative |  |
| 63 | None | No | Alive | Negative | Negative | Negative | Negative | Negative |  |
| 64 | None | No | Alive | Positive | Negative | Negative | 1:160 | Negative |  |
| 65 | None | No | Alive | Negative | Negative | Negative | 1:160 | Negative |  |
| 66 | None | No | Alive | Positive | Negative | Negative | 1:160 | Negative |  |
| 67 | None | Yes | Alive | Positive | Negative | Negative | 1:160 | Positive | Yes |
| 68 | None | No | Alive | Positive | Negative | Negative | 1:160 | Positive | Yes |
| 69 | None | No | Alive | Negative | Positive | Negative | Negative | Negative |  |
| 70 | None | No | Alive | Negative | Negative | Negative | 1:80 | Negative |  |
| 71 | Esophageal atresia | No | Alive | Positive | Positive | Negative | 1:40 | Negative |  |
| 72 | None | No | Alive | Negative | Negative | Negative | Negative | Negative |  |
| 73 | None | No | Alive | Positive | Negative | Positive | 1:80 | Negative |  |
| 74 | None | No | Alive | Positive | Negative | Negative | 1:160 | Positive | Yes |
| 75 | None | No | Alive | Negative | Negative | Negative | Negative | Negative |  |
| 76 | None | No | Alive | Positive | Negative | Negative | Negative | Negative |  |
| 77 | None | No | Alive | Negative | Positive | Negative | Negative | Positive | Yes |
| 78 | None | No | Alive | Negative | Negative | Negative | Negative | Negative |  |
| 79 | None | No | Alive | Positive | Negative | Negative | Negative | Negative |  |
| 80 | None | No | Alive | Negative | Negative | Negative | 1:40 | Negative |  |
| 81 | Brain lesions | No | Alive | Negative | Positive | Negative | Negative | Negative |  |
| 82 | None | No | Alive | Negative | Positive | Negative | 1:40 | Negative |  |
| 83 | None | No | Alive | Negative | Positive | Negative | Negative | Negative |  |
| 84 | None | No | Alive | Positive | Negative | Negative | 1:160 | Negative |  |
| 85 | Congenital heart disease | No | Alive | Negative | Negative | Negative | Negative | Negative |  |
| 86 | None | No | Alive | Negative | Negative | Negative | 1:160 | Negative |  |
| 87 | Congenital heart disease | No | Alive | Negative | Negative | Negative | Negative | Negative |  |
| 88 | None | Yes | Alive | Negative | Positive | Negative | 1:40 | Negative |  |
| 89 | None | No | Alive | Negative | Negative | Negative | Negative | Negative |  |
| 90 | None | No | Alive | Negative | Negative | Negative | Negative | Negative |  |
| 91 | None | No | Alive | Positive | Positive | Negative | 1:160 | Negative |  |
| 92 | None | No | Alive | Negative | Positive | Negative | 1:80 | Negative |  |
| 93 | None | No | Alive | Positive | Negative | Positive | 1:160 | Positive | Yes |
| 94 | None | No | Alive | Positive | Positive | Negative | 1:160 | Positive | Yes |
| 95 | None | No | Alive | Negative | Positive | Negative | 1:80 | Negative |  |
| 96 | None | No | Alive | Positive | Negative | Positive | 1:160 | Negative |  |
| 97 | None | No | Alive | Negative | Negative | Negative | Negative | Negative |  |
| 98 | None | No | Alive | Negative | Negative | Negative | Negative | Negative |  |
| 99 | None | No | Alive | Negative | Negative | Negative | Negative | Negative |  |
| 100 | None | No | Alive | Positive | Negative | Positive | 1:160 | Positive | Yes |
| 101 | None | No | Alive | Positive | Negative | Positive | 1:160 | Positive | Yes |
| 102 | None | No | Alive | Negative | Negative | Negative | 1:40 | Negative |  |
| 103 | None | No | Alive | Negative | Negative | Negative | 1:80 | Negative |  |
| 104 | None | No | Alive | Negative | Negative | Negative | Negative | Negative |  |
| 105 | None | No | Alive | Negative | Negative | Negative | Negative | Negative |  |
| 106 | None | No | Alive | Negative | Negative | Negative | 1:40 | Negative |  |
| 107 | Congenital heart disease | No | Alive | Negative | Negative | Negative | Negative | Negative |  |
| 108 | None | No | Alive | Positive | Negative | Negative | 1:80 | Negative |  |
| 109 | None | No | Alive | Positive | Negative | Negative | 1:160 | Positive | Yes |
| 110 | None | No | Alive | Negative | Positive | Negative | Negative | Negative |  |
| 111 | None | No | Alive | Positive | Positive | Negative | Negative | Positive | Yes |
| 112 | Bone fibroma | No | Alive | Positive | Negative | Negative | 1:160 | Positive | Yes |
| 113 | None | No | Alive | Negative | Negative | Negative | Negative | Negative |  |
| 114 | None | No | Alive | Positive | Negative | Negative | 1:160 | Positive | Yes |
| 115 | None | No | Alive | Positive | Negative | Positive | 1:160 | Positive | Yes |
| 116 | None | Yes | Alive | Positive | Positive | Negative | 1:160 | Positive | Yes |
| 117 | None | No | Alive | Positive | Positive | Negative | 1:160 | Positive | Yes |
| 118 | None | No | Alive | Negative | Negative | Negative | 1:160 | Negative |  |
| 119 | None | No | Alive | Positive | Negative | Positive | 1:160 | Positive | Yes |
| 120 | None | No | Alive | Positive | Negative | Negative | 1:160 | Positive | Yes |
| 121 | None | No | Alive | Negative | Negative | Negative | Negative | Negative |  |
| 122 | Congenital heart disease | No | Alive | Positive | Positive | Negative | Negative | Negative |  |
| 123 | None | No | Alive | Positive | Negative | Positive | 1:160 | Negative |  |
| 124 | None | No | Alive | Positive | Negative | Negative | Negative | Negative |  |
| 125 | Brain lesions | No | Alive | Negative | Positive | Negative | Negative | Negative |  |
| 126 | None | No | Alive | Positive | Negative | Negative | 1:160 | Negative |  |
| 127 | None | No | Alive | Positive | Positive | Positive | Negative | Positive | Yes |
| 128 | None | No | Alive | Negative | Negative | Negative | Negative | Negative |  |
| 129 | None | No | Alive | Negative | Negative | Negative | Negative | Negative |  |
| 130 | None | No | Alive | Positive | Negative | Positive | 1:160 | Positive | Yes |
| 131 | Brain lesions | No | Alive | Negative | Positive | Negative | Negative | Negative |  |
| 132 | None | No | Alive | Positive | Negative | Negative | 1:160 | Negative |  |
| 133 | None | No | Alive | Positive | Negative | Negative | 1:160 | Negative |  |
| 134 | Congenital heart disease | No | Alive | Negative | Negative | Negative | Negative | Negative |  |
| 135 | None | No | Alive | Negative | Negative | Negative | 1:40 | Negative |  |
| 136 | None | No | Alive | Positive | Negative | Negative | 1:160 | Negative |  |
| 137 | Congenital heart disease | No | Alive | Negative | Negative | Negative | Negative | Negative |  |
| 138 | None | No | Alive | Negative | Positive | Negative | Negative | Negative |  |
| 139 | Congenital heart disease | No | Alive | Negative | Positive | Negative | Negative | Negative |  |
| 140 | None | No | Alive | Positive | Negative | Negative | 1:160 | Negative |  |
| 141 | Congenital heart disease | Yes | Alive | Negative | Positive | Negative | 1:40 | Negative |  |
| 142 | None | No | Alive | Positive | Negative | Negative | 1:160 | Negative |  |
| 143 | None | No | Alive | Negative | Negative | Negative | Negative | Negative |  |
| 144 | Esophageal atresia | No | Alive | Positive | Positive | Negative | 1:40 | Negative |  |
| 145 | None | No | Alive | Negative | Negative | Negative | 1:40 | Negative |  |
| 146 | None | Yes | Alive | Positive | Negative | Negative | 1:160 | Positive | Yes |
| 147 | Brain lesions | No | Alive | Negative | Positive | Negative | Negative | Negative |  |
| 148 | None | No | Alive | Negative | Negative | Negative | 1:80 | Negative |  |
| 149 | None | No | Alive | Positive | Negative | Negative | 1:160 | Negative |  |
| 150 | None | No | Alive | Negative | Negative | Negative | 1:40 | Positive | No |
| 151 | None | Yes | Alive | Negative | Negative | Negative | Negative | Negative |  |
| 152 | None | No | Alive | Negative | Negative | Negative | 1:160 | Positive | Yes |
| 153 | None | No | Alive | Negative | Negative | Negative | Negative | Negative |  |
| 154 | None | No | Alive | Negative | Positive | Negative | 1:40 | Negative |  |
| 155 | None | No | Alive | Negative | Negative | Negative | 1:160 | Negative |  |
| 156 | None | No | Alive | Positive | Negative | Positive | 1:160 | Positive | Yes |
| 157 | None | No | Alive | Positive | Negative | Positive | 1:160 | Negative |  |
| 158 | None | No | Alive | Negative | Positive | Negative | Negative | Negative |  |
| 159 | None | No | Alive | Negative | Negative | Negative | Negative | Negative |  |
| 160 | None | No | Alive | Positive | Negative | Negative | 1:640 | Positive | Yes |
| 161 | Congenital heart disease | Yes | Alive | Negative | Negative | Negative | Negative | Negative |  |
| 162 | None | No | Alive | Negative | Negative | Positive | Negative | Positive | Yes |
| 163 | None | No | Alive | Positive | Negative | Negative | 1:160 | Positive | Yes |
| 164 | None | No | Alive | Negative | Negative | Negative | Negative | Positive | Yes |
| 165 | None | Yes | Alive | Positive | Negative | Positive | 1:160 | Positive | Yes |
| 166 | None | No | Alive | Negative | Negative | Negative | Negative | Negative |  |
| 167 | None | No | Alive | Positive | Positive | Negative | 1:160 | Positive | Yes |
| 168 | None | No | Alive | Positive | Negative | Positive | 1:80 | Negative |  |
| 169 | None | No | Alive | Negative | Negative | Negative | 1:160 | Positive | Yes |
| 170 | None | No | Alive | Negative | Negative | Negative | Negative | Negative |  |
| 171 | None | No | Alive | Negative | Positive | Negative | Negative | Negative |  |
| 172 | None | No | Alive | Negative | Negative | Negative | 1:40 | Negative |  |
| 173 | None | No | Alive | Negative | Positive | Negative | Negative | Negative |  |
| 174 | None | No | Alive | Negative | Negative | Negative | Negative | Negative |  |
| 175 | None | No | Alive | Negative | Negative | Negative | Negative | Negative |  |
| 176 | None | No | Alive | Negative | Negative | Negative | Negative | Negative |  |
| 177 | None | No | Alive | Negative | Negative | Negative | Negative | Negative |  |
| 178 | None | No | Alive | Negative | Negative | Negative | 1:160 | Negative |  |
| 179 | None | No | Alive | Positive | Negative | Negative | 1:160 | Negative |  |
| 180 | None | No | Alive | Negative | Negative | Negative | Negative | Positive | Yes |
| 181 | None | No | Alive | Negative | Negative | Negative | Negative | Negative |  |
| 182 | None | No | Alive | Negative | Negative | Negative | Negative | Negative |  |
| 183 | None | No | Alive | Negative | Positive | Negative | 1:160 | Negative |  |
| 184 | None | No | Alive | Negative | Negative | Negative | 1:80 | Negative |  |
| 185 | None | No | Alive | Negative | Negative | Negative | Negative | Negative |  |
| 186 | None | No | Alive | Negative | Negative | Negative | Negative | Negative |  |
| 187 | None | No | Alive | Positive | Negative | Negative | 1:160 | Positive | No |
| 188 | None | No | Alive | Positive | Negative | Negative | 1:160 | Positive | Yes |
| 189 | None | No | Alive | Negative | Negative | Negative | 1:80 | Negative |  |
| 190 | None | No | Alive | Negative | Negative | Negative | Negative | Negative |  |
| 191 | None | No | Alive | Negative | Positive | Negative | 1:160 | Positive | Yes |
| 192 | None | No | Alive | Negative | Negative | Negative | 1:80 | Negative |  |
| 193 | None | No | Alive | Negative | Negative | Negative | Negative | Negative |  |
| 194 | None | No | Alive | Negative | Negative | Negative | Negative | Negative |  |
| 195 | None | No | Alive | Positive | Negative | Negative | 1:160 | Negative |  |
| 196 | Congenital heart disease | No | Alive | Negative | Negative | Negative | 1:40 | Negative |  |
| 197 | None | No | Alive | Negative | Negative | Positive | 1:160 | Positive | Yes |
| 198 | Congenital biliary atresia | No | Alive | Negative | Negative | Negative | 1:80 | Negative |  |
| 199 | None | No | Alive | Positive | Negative | Negative | 1:160 | Negative |  |
| 200 | None | No | Alive | Negative | Negative | Negative | Negative | Negative |  |
| 201 | None | No | Alive | Negative | Negative | Negative | 1:160 | Negative |  |
| 202 | None | No | Alive | Negative | Negative | Negative | Negative | Negative |  |
| 203 | None | No | Alive | Negative | Negative | Negative | Negative | Positive | No |
| 204 | None | No | Alive | Negative | Negative | Negative | Negative | Negative |  |
| 205 | Congenital heart disease | No | Alive | Negative | Negative | Negative | 1:80 | Negative |  |
| 206 | None | No | Alive | Negative | Negative | Negative | Negative | Negative |  |
| 207 | None | No | Alive | Positive | Negative | Negative | 1:160 | Negative |  |
| 208 | None | No | Alive | Negative | Negative | Negative | Negative | Negative |  |
| 209 | None | No | Alive | Negative | Negative | Negative | Negative | Negative |  |
| 210 | None | No | Alive | Negative | Positive | Positive | Negative | Positive | Yes |
| 211 | None | No | Alive | Negative | Negative | Negative | 1:160 | Positive | Yes |
| 212 | None | No | Alive | Negative | Positive | Negative | Negative | Positive | No |
| 213 | None | No | Alive | Negative | Negative | Negative | Negative | Negative |  |
| 214 | None | No | Alive | Negative | Negative | Negative | Negative | Negative |  |
| 215 | Esophageal atresia | No | Alive | Negative | Negative | Negative | 1:160 | Negative |  |
| 216 | None | No | Alive | Negative | Negative | Negative | Negative | Negative |  |
| 217 | None | No | Alive | Negative | Positive | Negative | Negative | Negative |  |
| 218 | None | No | Alive | Positive | Positive | Positive | 1:160 | Positive | Yes |
| 219 | None | No | Alive | Negative | Negative | Negative | 1:80 | Negative |  |
| 220 | None | No | Alive | Positive | Negative | Negative | 1:160 | Positive | Yes |
| 221 | None | No | Alive | Negative | Negative | Negative | 1:40 | Negative |  |
| 222 | Congenital heart disease | Yes | Alive | Negative | Negative | Negative | Negative | Negative |  |
| 223 | None | No | Alive | Negative | Negative | Negative | Negative | Negative |  |
| 224 | None | No | Alive | Negative | Negative | Negative | 1:160 | Positive | No |
| 225 | None | No | Alive | Negative | Negative | Negative | 1:160 | Negative |  |
| 226 | None | No | Alive | Positive | Negative | Negative | 1:160 | Negative |  |
| 227 | None | No | Alive | Negative | Negative | Negative | Negative | Negative |  |
| 228 | None | No | Alive | Positive | Negative | Negative | 1:160 | Positive | Yes |
| 229 | None | No | Alive | Positive | Negative | Negative | 1:160 | Negative |  |
| 230 | None | No | Alive | Negative | Negative | Negative | 1:40 | Negative |  |
| 231 | None | No | Alive | Positive | Negative | Negative | 1:160 | Positive | Yes |
| 232 | None | No | Alive | Negative | Negative | Negative | 1:80 | Negative |  |
| 233 | None | No | Alive | Positive | Negative | Negative | 1:160 | Negative |  |
| 234 | None | No | Alive | Negative | Negative | Negative | Negative | Negative |  |
| 235 | None | No | Alive | Negative | Negative | Negative | Negative | Negative |  |
| 236 | None | No | Alive | Negative | Negative | Positive | 1:40 | Positive | No |
| 237 | None | No | Alive | Positive | Negative | Negative | 1:80 | Negative |  |
| 238 | None | No | Alive | Positive | Negative | Negative | 1:80 | Positive | Yes |
| 239 | None | No | Alive | Negative | Negative | Negative | Negative | Negative |  |
| 240 | None | No | Alive | Negative | Negative | Negative | 1:40 | Negative |  |
| 241 | None | No | Alive | Negative | Negative | Positive | Negative | Positive | Yes |
| 242 | None | No | Alive | Negative | Negative | Negative | 1:80 | Negative |  |
| 243 | None | No | Alive | Negative | Negative | Negative | Negative | Negative |  |
| 244 | None | No | Alive | Negative | Negative | Negative | Negative | Negative |  |
| 245 | None | No | Alive | Negative | Negative | Negative | 1:80 | Negative |  |
| 246 | Congenital heart disease | No | Alive | Negative | Negative | Negative | Negative | Negative |  |
| 247 | None | No | Alive | Negative | Negative | Negative | Negative | Negative |  |
| 248 | None | No | Alive | Positive | Negative | Positive | 1:160 | Positive | Yes |
| 249 | Congenital heart disease | Yes | Death | Negative | Negative | Negative | Negative | Negative |  |
| 250 | None | No | Alive | Positive | Negative | Negative | 1:160 | Positive | Yes |
| 251 | None | No | Alive | Positive | Negative | Negative | 1:160 | Positive | Yes |
| 252 | None | No | Alive | Negative | Negative | Negative | Negative | Negative |  |
| 253 | None | No | Alive | Positive | Negative | Negative | 1:160 | Positive | Yes |
| 254 | None | No | Alive | Positive | Negative | Negative | 1:160 | Negative |  |
| 255 | None | No | Alive | Negative | Negative | Negative | 1:160 | Negative |  |
| 256 | None | No | Alive | Positive | Negative | Negative | 1:160 | Positive | Yes |
| 257 | None | No | Alive | Positive | Negative | Positive | 1:160 | Positive | Yes |
| 258 | None | No | Alive | Negative | Negative | Negative | Negative | Negative |  |
| 259 | None | No | Alive | Positive | Negative | Negative | 1:160 | Positive | Yes |
| 260 | None | No | Alive | Negative | Negative | Negative | Negative | Negative |  |
| 261 | None | No | Alive | Negative | Negative | Negative | Negative | Negative |  |
| 262 | None | No | Alive | Negative | Negative | Negative | Negative | Negative |  |
| 263 | None | No | Alive | Negative | Negative | Negative | 1:40 | Negative |  |
| 264 | None | No | Alive | Negative | Negative | Negative | 1:80 | Negative |  |
| 265 | None | No | Alive | Negative | Negative | Negative | Negative | Negative |  |
| 266 | None | No | Alive | Negative | Negative | Negative | 1:160 | Negative |  |
| 267 | None | Yes | Alive | Positive | Positive | Negative | 1:160 | Positive | Yes |
| 268 | Congenital heart disease | No | Alive | Negative | Negative | Negative | 1:80 | Positive | Yes |
| 269 | None | No | Alive | Negative | Negative | Negative | Negative | Negative |  |
| 270 | None | No | Alive | Negative | Negative | Negative | Negative | Negative |  |
| 271 | Congenital heart disease | No | Alive | Negative | Negative | Negative | Negative | Negative |  |
| 272 | None | No | Alive | Negative | Negative | Negative | Negative | Negative |  |
| 273 | None | No | Alive | Positive | Negative | Negative | 1:160 | Positive | Yes |
| 274 | Tracheal stenosis | No | Alive | Negative | Negative | Negative | Negative | Negative |  |
| 275 | Tracheal stenosis | No | Alive | Negative | Negative | Negative | Negative | Negative |  |
| 276 | None | No | Alive | Negative | Negative | Negative | Negative | Negative |  |
| 277 | None | No | Alive | Negative | Negative | Negative | 1:40 | Negative |  |
| 278 | None | No | Alive | Negative | Negative | Negative | Negative | Negative |  |
| 279 | None | No | Alive | Negative | Negative | Negative | 1:40 | Positive | Yes |
| 280 | None | No | Alive | Negative | Negative | Negative | Negative | Negative |  |
| 281 | None | No | Alive | Negative | Negative | Negative | Negative | Negative |  |
| 282 | None | No | Alive | Negative | Negative | Negative | Negative | Negative |  |
| 283 | None | No | Alive | Negative | Negative | Negative | 1:40 | Negative |  |
| 284 | None | No | Alive | Negative | Negative | Negative | Negative | Negative |  |
| 285 | None | No | Alive | Positive | Negative | Negative | 1:160 | Positive | Yes |
| 286 | None | No | Alive | Negative | Negative | Negative | 1:160 | Positive | Yes |
| 287 | None | No | Alive | Negative | Negative | Negative | Negative | Negative |  |
| 288 | None | No | Alive | Negative | Negative | Negative | Negative | Negative |  |
| 289 | None | No | Alive | Negative | Positive | Positive | 1:160 | Positive | Yes |
| 290 | Brain lesions | No | Alive | Negative | Negative | Negative | Negative | Negative |  |
| 291 | None | No | Alive | Negative | Negative | Negative | Negative | Negative |  |
| 292 | None | No | Alive | Negative | Negative | Negative | 1:40 | Negative |  |
| 293 | Congenital heart disease | No | Alive | Negative | Negative | Negative | Negative | Negative |  |
| 294 | None | No | Alive | Negative | Negative | Negative | Negative | Negative |  |
| 295 | None | No | Alive | Negative | Positive | Negative | 1:40 | Positive | Yes |
| 296 | None | No | Alive | Negative | Negative | Negative | 1:40 | Negative |  |
| 297 | None | No | Alive | Negative | Negative | Negative | Negative | Negative |  |
| 298 | None | No | Alive | Negative | Negative | Negative | Negative | Negative |  |
| 299 | None | No | Alive | Negative | Negative | Negative | 1:40 | Negative |  |
| 300 | None | No | Alive | Negative | Negative | Negative | Negative | Negative |  |
| 301 | None | No | Alive | Negative | Negative | Negative | Negative | Negative |  |
| 302 | None | No | Alive | Negative | Negative | Negative | 1:80 | Positive | No |
| 303 | None | No | Alive | Negative | Negative | Negative | 1:40 | Negative |  |
| 304 | None | No | Alive | Negative | Negative | Negative | Negative | Negative |  |
| 305 | None | No | Alive | Negative | Negative | Negative | Negative | Negative |  |
| 306 | None | No | Alive | Negative | Positive | Negative | 1:80 | Positive | Yes |
| 307 | None | No | Alive | Negative | Negative | Negative | Negative | Negative |  |
| 308 | None | No | Alive | Negative | Negative | Negative | 1:40 | Negative |  |
| 309 | None | No | Alive | Negative | Negative | Negative | 1:40 | Negative |  |
| 310 | None | No | Alive | Negative | Negative | Negative | Negative | Negative |  |
| 311 | None | No | Alive | Negative | Negative | Negative | Negative | Negative |  |
| 312 | None | No | Alive | Negative | Negative | Negative | 1:160 | Negative |  |
| 313 | None | No | Alive | Negative | Negative | Negative | Negative | Negative |  |
| 314 | None | No | Alive | Negative | Negative | Negative | Negative | Negative |  |
| 315 | None | No | Alive | Negative | Negative | Negative | Negative | Negative |  |
| 316 | None | No | Alive | Negative | Negative | Negative | Negative | Negative |  |
| 317 | None | No | Alive | Negative | Negative | Negative | Negative | Negative |  |
| 318 | None | No | Alive | Negative | Negative | Negative | Negative | Negative |  |
| 319 | None | No | Alive | Negative | Negative | Negative | 1:40 | Negative |  |
| 320 | None | No | Alive | Positive | Negative | Negative | 1:160 | Positive | Yes |
| 321 | None | No | Alive | Negative | Negative | Negative | 1:40 | Negative |  |
| 322 | Congenital heart disease | No | Alive | Negative | Negative | Negative | Negative | Negative |  |
| 323 | None | No | Alive | Negative | Negative | Negative | Negative | Positive | No |
| 324 | Congenital heart disease | No | Alive | Negative | Negative | Negative | 1:160 | Negative |  |
| 325 | None | No | Alive | Negative | Negative | Positive | 1:160 | Positive | Yes |
| 326 | Congenital heart disease | No | Alive | Negative | Negative | Negative | Negative | Negative |  |
| 327 | None | No | Alive | Negative | Negative | Negative | Negative | Negative |  |
| 328 | Congenital heart disease | No | Alive | Negative | Negative | Negative | 1:160 | Negative |  |
| 329 | None | No | Alive | Negative | Negative | Negative | Negative | Negative |  |
| 330 | None | No | Alive | Negative | Negative | Negative | 1:80 | Negative |  |
| 331 | None | No | Alive | Positive | Negative | Negative | 1:160 | Positive | Yes |
| 332 | None | No | Alive | Negative | Negative | Negative | Negative | Negative |  |
| 333 | None | No | Alive | Negative | Negative | Negative | Negative | Negative |  |
| 334 | None | No | Alive | Negative | Negative | Negative | Negative | Negative |  |
| 335 | None | No | Alive | Negative | Negative | Negative | 1:80 | Negative |  |
| 336 | None | No | Alive | Negative | Negative | Negative | Negative | Negative |  |
| 337 | None | No | Alive | Negative | Negative | Negative | Negative | Negative |  |
| 338 | None | No | Alive | Negative | Negative | Negative | 1:80 | Negative |  |
| 339 | None | No | Alive | Positive | Negative | Negative | 1:160 | Negative |  |
| 340 | Congenital heart disease | No | Alive | Negative | Negative | Negative | Negative | Positive | Yes |
| 341 | Congenital heart disease | No | Alive | Negative | Negative | Negative | Negative | Negative |  |
| 342 | None | No | Alive | Negative | Negative | Negative | Negative | Negative |  |
| 343 | Congenital heart disease | No | Alive | Negative | Negative | Negative | Negative | Negative |  |
| 344 | None | No | Alive | Positive | Positive | Negative | 1:160 | Positive | Yes |
| 345 | None | No | Alive | Negative | Negative | Negative | Negative | Negative |  |
| 346 | Congenital heart disease | Yes | Alive | Negative | Negative | Negative | Negative | Negative |  |
| 347 | None | No | Alive | Positive | Negative | Negative | 1:160 | Positive | Yes |
| 348 | None | No | Alive | Negative | Negative | Negative | Negative | Positive | Yes |
| 349 | Brain lesions | No | Alive | Negative | Negative | Negative | Negative | Negative |  |
| 350 | None | No | Alive | Positive | Positive | Positive | 1:160 | Positive | Yes |
| 351 | None | No | Alive | Positive | Positive | Negative | 1:160 | Positive | Yes |
| 352 | None | No | Alive | Negative | Negative | Negative | 1:40 | Negative |  |
| 353 | None | No | Alive | Negative | Negative | Negative | Negative | Negative |  |
| 354 | None | No | Alive | Positive | Negative | Negative | 1:160 | Negative |  |
| 355 | None | No | Alive | Positive | Negative | Negative | 1:160 | Negative |  |
| 356 | Congenital heart disease | No | Alive | Negative | Negative | Negative | Negative | Negative |  |
| 357 | Congenital biliary atresia | Yes | Alive | Negative | Negative | Negative | Negative | Negative |  |
| 358 | Congenital heart disease | No | Alive | Negative | Negative | Negative | Negative | Negative |  |
| 359 | None | No | Alive | Negative | Negative | Negative | Negative | Negative |  |
| 360 | None | No | Alive | Negative | Negative | Negative | Negative | Negative |  |
| 361 | None | No | Alive | Positive | Negative | Negative | 1:160 | Positive | Yes |
| 362 | None | No | Alive | Positive | Negative | Negative | 1:80 | Positive | Yes |
| 363 | primary immunodeficiency disease | Yes | Death | Positive | Negative | Negative | 1:80 | Negative |  |
| 364 | None | No | Alive | Positive | Positive | Negative | 1:80 | Positive | Yes |
| 365 | None | No | Alive | Negative | Negative | Negative | Negative | Negative |  |
| 366 | None | No | Alive | Positive | Negative | Positive | 1:160 | Positive | Yes |
| 367 | None | No | Alive | Positive | Negative | Negative | 1:160 | Positive | Yes |
| 368 | None | No | Alive | Positive | Negative | Negative | 1:160 | Negative |  |
| 369 | None | No | Alive | Negative | Positive | Positive | Negative | Positive | Yes |
| 370 | None | No | Alive | Positive | Negative | Positive | 1:80 | Positive | Yes |
| 371 | Congenital heart disease | No | Alive | Positive | Negative | Negative | 1:160 | Positive | Yes |
| 372 | None | No | Alive | Positive | Negative | Negative | 1:160 | Positive | Yes |
| 373 | Congenital heart disease | No | Alive | Negative | Negative | Negative | Negative | Negative |  |
| 374 | None | No | Alive | Negative | Negative | Negative | Negative | Negative |  |
| 375 | None | No | Alive | Negative | Negative | Negative | Negative | Negative |  |
| 376 | None | No | Alive | Positive | Negative | Negative | 1:160 | Positive | Yes |
| 377 | Brain lesions | No | Alive | Negative | Negative | Negative | Negative | Negative |  |
| 378 | None | No | Alive | Negative | Negative | Negative | Negative | Negative |  |
| 379 | None | No | Alive | Negative | Negative | Negative | Negative | Negative |  |
| 380 | None | No | Alive | Positive | Negative | Negative | 1:160 | Negative |  |
| 381 | None | No | Alive | Negative | Positive | Negative | 1:160 | Positive | Yes |
| 382 | None | No | Alive | Positive | Negative | Negative | 1:160 | Positive | Yes |
| 383 | None | No | Alive | Negative | Negative | Negative | Negative | Negative |  |
| 384 | None | No | Alive | Negative | Negative | Negative | Negative | Negative |  |
| 385 | None | No | Alive | Positive | Positive | Positive | 1:160 | Positive | Yes |
| 386 | None | No | Alive | Negative | Negative | Negative | Negative | Negative |  |
| 387 | None | No | Alive | Negative | Negative | Negative | 1:160 | Negative |  |
| 388 | Congenital heart disease | No | Alive | Negative | Negative | Negative | Negative | Negative |  |
| 389 | None | No | Alive | Negative | Negative | Negative | 1:80 | Negative |  |
| 390 | Congenital heart disease | Yes | Alive | Positive | Negative | Positive | 1:160 | Positive | Yes |
| 391 | None | No | Alive | Positive | Positive | Positive | 1:160 | Positive | Yes |
| 392 | None | No | Alive | Negative | Negative | Negative | Negative | Negative |  |
| 393 | None | No | Alive | Negative | Negative | Negative | 1:160 | Positive | Yes |
| 394 | None | No | Alive | Negative | Negative | Negative | 1:160 | Negative |  |
| 395 | None | Yes | Alive | Positive | Negative | Positive | 1:160 | Positive | Yes |
| 396 | None | No | Alive | Positive | Negative | Positive | 1:160 | Positive | Yes |
| 397 | None | No | Alive | Negative | Negative | Positive | 1:80 | Negative |  |
| 398 | None | No | Alive | Negative | Negative | Negative | 1:80 | Negative |  |
| 399 | None | No | Alive | Positive | Negative | Negative | 1:160 | Positive | Yes |
| 400 | Congenital biliary atresia | No | Alive | Negative | Negative | Negative | Negative | Positive | Yes |
| 401 | None | No | Alive | Positive | Positive | Negative | 1:160 | Positive | Yes |
| 402 | None | No | Alive | Negative | Negative | Negative | 1:160 | Negative |  |
| 403 | None | Yes | Alive | Negative | Negative | Negative | Negative | Negative |  |
| 404 | None | No | Alive | Negative | Positive | Positive | 1:160 | Positive | Yes |
| 405 | Congenital heart disease | No | Alive | Negative | Negative | Negative | Negative | Negative |  |
| 406 | Tracheal stenosis | No | Alive | Negative | Negative | Negative | Negative | Negative |  |
| 407 | None | No | Alive | Negative | Negative | Negative | Negative | Negative |  |
| 408 | None | No | Alive | Positive | Negative | Negative | 1:160 | Positive | Yes |
| 409 | None | No | Alive | Negative | Negative | Positive | 1:160 | Positive | Yes |
| 410 | Congenital heart disease | Yes | Alive | Negative | Negative | Negative | Negative | Negative |  |
| 411 | None | No | Alive | Negative | Negative | Negative | 1:160 | Negative |  |
| 412 | None | No | Alive | Negative | Negative | Negative | 1:40 | Negative |  |
| 413 | Congenital heart disease | No | Alive | Negative | Negative | Negative | Negative | Negative |  |
| 414 | None | No | Alive | Negative | Negative | Negative | Negative | Negative |  |
| 415 | Congenital heart disease | Yes | Alive | Negative | Negative | Negative | Negative | Negative |  |
| 416 | None | No | Alive | Negative | Negative | Negative | Negative | Negative |  |
| 417 | Congenital heart disease | No | Alive | Negative | Negative | Negative | Negative | Negative |  |
| 418 | None | Yes | Alive | Negative | Negative | Negative | 1:40 | Positive | No |
| 419 | None | No | Alive | Positive | Negative | Negative | 1:160 | Positive | Yes |
| 420 | None | No | Alive | Negative | Negative | Negative | 1:40 | Negative |  |
| 421 | None | No | Alive | Positive | Negative | Positive | 1:160 | Positive | Yes |
| 422 | Congenital heart disease | No | Alive | Negative | Negative | Negative | Negative | Negative |  |
| 423 | None | Yes | Alive | Negative | Negative | Negative | 1:40 | Negative |  |
| 424 | None | No | Alive | Positive | Positive | Negative | 1:160 | Positive | Yes |
| 425 | Congenital heart disease | No | Alive | Negative | Negative | Negative | Negative | Negative |  |
| 426 | None | No | Alive | Positive | Negative | Negative | 1:160 | Positive | Yes |
| 427 | None | No | Alive | Positive | Negative | Positive | 1:160 | Positive | Yes |
| 428 | None | No | Alive | Negative | Negative | Negative | 1:80 | Negative |  |
| 429 | None | Yes | Alive | Positive | Positive | Positive | 1:160 | Positive | Yes |
| 430 | None | No | Alive | Negative | Negative | Negative | 1:160 | Positive | Yes |
| 431 | Congenital heart disease | No | Alive | Negative | Negative | Negative | Negative | Negative |  |
| 432 | None | No | Alive | Negative | Negative | Negative | Negative | Negative |  |
| 433 | None | No | Alive | Negative | Negative | Negative | Negative | Negative |  |
| 434 | None | Yes | Alive | Negative | Negative | Negative | Negative | Negative |  |
| 435 | Congenital heart disease | Yes | Alive | Negative | Negative | Negative | Negative | Positive | Yes |
| 436 | None | No | Alive | Positive | Negative | Negative | 1:80 | Positive | No |
| 437 | Congenital heart disease | No | Alive | Negative | Negative | Negative | 1:40 | Positive | No |
| 438 | Brain lesions | Yes | Alive | Negative | Negative | Negative | Negative | Negative |  |
| 439 | Congenital heart disease | No | Alive | Negative | Negative | Negative | 1:160 | Positive | No |
| 440 | Congenital heart disease | No | Alive | Negative | Negative | Negative | Negative | Negative |  |
| 441 | None | No | Alive | Negative | Negative | Negative | Negative | Negative |  |
| 442 | None | No | Alive | Negative | Negative | Negative | 1:160 | Positive | Yes |
| 443 | Brain lesions | Yes | Alive | Negative | Negative | Negative | 1:80 | Negative |  |
| 444 | None | No | Alive | Negative | Positive | Negative | 1:80 | Positive | No |
| 445 | Congenital heart disease | No | Alive | Negative | Negative | Negative | Negative | Negative |  |
| 446 | None | No | Alive | Negative | Negative | Negative | 1:160 | Positive | No |
| 447 | None | No | Alive | Negative | Negative | Negative | Negative | Negative |  |
| 448 | Congenital heart disease | No | Alive | Negative | Negative | Negative | Negative | Positive | No |
| 449 | None | No | Alive | Negative | Negative | Negative | 1:40 | Negative |  |
| 450 | None | No | Alive | Negative | Negative | Negative | Negative | Negative |  |
| 451 | None | No | Alive | Negative | Negative | Negative | Negative | Negative |  |
| 452 | None | No | Alive | Negative | Positive | Positive | 1:40 | Positive | Yes |
| 453 | None | No | Alive | Negative | Negative | Negative | Negative | Negative |  |
| 454 | None | Yes | Alive | Positive | Positive | Positive | 1:160 | Positive | Yes |
| 455 | None | No | Alive | Negative | Negative | Negative | 1:160 | Negative |  |
| 456 | None | No | Alive | Negative | Negative | Negative | Negative | Negative |  |
| 457 | None | No | Alive | Negative | Negative | Positive | 1:640 | Positive | Yes |
| 458 | None | Yes | Alive | Negative | Negative | Negative | Negative | Negative |  |
| 459 | None | No | Alive | Positive | Negative | Positive | 1:640 | Positive | Yes |
| 460 | Congenital heart disease | Yes | Alive | Negative | Negative | Negative | Negative | Negative |  |
| 461 | None | No | Alive | Negative | Negative | Negative | 1:640 | Positive | Yes |
| 462 | Congenital heart disease | Yes | Alive | Negative | Negative | Negative | 1:40 | Negative |  |
| 463 | Congenital heart disease | Yes | Alive | Negative | Negative | Negative | 1:40 | Negative |  |
| 464 | Brain lesions | No | Alive | Negative | Negative | Negative | 1:160 | Positive | Yes |
| 465 | None | No | Alive | Negative | Negative | Negative | Negative | Negative |  |
| 466 | None | No | Alive | Positive | Negative | Negative | 1:320 | Positive | Yes |
| 467 | None | No | Alive | Negative | Positive | Positive | 1:80 | Positive | Yes |
| 468 | None | No | Alive | Negative | Negative | Negative | Negative | Negative |  |
| 469 | None | No | Alive | Negative | Negative | Negative | 1:40 | Negative |  |
| 470 | None | No | Alive | Negative | Negative | Negative | 1:40 | Negative |  |
| 471 | Congenital heart disease | No | Alive | Negative | Negative | Negative | 1:160 | Negative |  |
| 472 | None | No | Alive | Negative | Negative | Negative | 1:40 | Negative |  |
| 473 | Congenital heart disease | No | Alive | Negative | Negative | Negative | 1:80 | Negative |  |
| 474 | None | No | Alive | Negative | Negative | Negative | Negative | Negative |  |
| 475 | Nasal fistula | No | Alive | Negative | Negative | Negative | Negative | Negative |  |
| 476 | None | No | Alive | Positive | Negative | Negative | 1:640 | Positive | Yes |
| 477 | Acute lymphocyte leukocyte | No | Alive | Negative | Negative | Negative | 1:40 | Negative |  |
| 478 | None | No | Alive | Positive | Negative | Negative | 1:640 | Positive | Yes |
| 479 | None | No | Alive | Positive | Negative | Positive | 1:640 | Positive | Yes |
| 480 | Congenital heart disease | No | Alive | Negative | Negative | Negative | Negative | Negative |  |
| 481 | None | No | Alive | Positive | Negative | Negative | 1:80 | Negative |  |
| 482 | None | No | Alive | Negative | Negative | Negative | Negative | Negative |  |
| 483 | None | No | Alive | Negative | Negative | Positive | 1:160 | Positive | Yes |
| 484 | None | No | Alive | Positive | Negative | Negative | 1:640 | Positive | Yes |
| 485 | None | No | Alive | Negative | Negative | Negative | Negative | Negative |  |
| 486 | None | No | Alive | Negative | Negative | Negative | Negative | Negative |  |
| 487 | Hiatal hernia | No | Alive | Negative | Negative | Negative | 1:40 | Negative |  |
| 488 | None | No | Alive | Negative | Negative | Positive | 1:640 | Positive | Yes |
| 489 | None | No | Alive | Positive | Negative | Positive | 1:640 | Positive | No |
| 490 | None | No | Alive | Negative | Negative | Negative | 1:320 | Negative |  |
| 491 | Thoracic tumors | No | Alive | Positive | Negative | Negative | 1:640 | Negative |  |
| 492 | None | No | Alive | Negative | Negative | Positive | 1:320 | Positive | Yes |
| 493 | None | No | Alive | Negative | Negative | Negative | 1:160 | Negative |  |
| 494 | Congenital heart disease | No | Alive | Negative | Negative | Negative | 1:320 | Negative |  |
| 495 | None | No | Alive | Positive | Negative | Negative | 1:640 | Positive | Yes |
| 496 | None | No | Alive | Negative | Negative | Negative | Negative | Negative |  |
| 497 | None | No | Alive | Negative | Negative | Positive | Negative | Positive | No |
| 498 | None | No | Alive | Negative | Negative | Negative | 1:40 | Negative |  |
| 499 | None | No | Alive | Negative | Negative | Negative | Negative | Negative |  |
| 500 | Congenital heart disease | No | Alive | Negative | Positive | Negative | 1:80 | Positive | Yes |
| 501 | None | No | Alive | Negative | Negative | Negative | Negative | Negative |  |
| 502 | Congenital heart disease | No | Alive | Negative | Negative | Negative | 1:160 | Negative |  |
| 503 | None | No | Alive | Negative | Negative | Negative | Negative | Negative |  |
| 504 | None | No | Alive | Negative | Negative | Positive | 1:640 | Positive | Yes |
| 505 | None | No | Alive | Positive | Negative | Negative | 1:640 | Positive | Yes |
| 506 | Congenital heart disease | Yes | Alive | Negative | Negative | Negative | Negative | Negative |  |
| 507 | None | No | Alive | Negative | Negative | Negative | 1:40 | Negative |  |
| 508 | None | No | Alive | Positive | Positive | Positive | 1:640 | Positive | Yes |
| 509 | None | No | Alive | Negative | Negative | Negative | Negative | Negative |  |
| 510 | None | No | Alive | Negative | Negative | Negative | 1:640 | Positive | Yes |
| 511 | Brain lesions | No | Alive | Negative | Negative | Negative | Negative | Negative |  |
| 512 | Congenital heart disease | No | Alive | Negative | Negative | Negative | Negative | Negative |  |
| 513 | None | No | Alive | Negative | Positive | Positive | 1:640 | Positive | Yes |
| 514 | None | No | Alive | Negative | Negative | Negative | Negative | Negative |  |
| 515 | None | No | Alive | Positive | Positive | Negative | 1:160 | Positive | Yes |
| 516 | None | No | Alive | Positive | Positive | Positive | 1:640 | Positive | Yes |
| 517 | None | No | Alive | Negative | Negative | Negative | 1:640 | Negative |  |
| 518 | None | Yes | Alive | Negative | Negative | Negative | 1:320 | Negative |  |
| 519 | None | No | Alive | Negative | Positive | Negative | Negative | Negative |  |
| 520 | None | No | Alive | Positive | Negative | Positive | 1:640 | Positive | Yes |
| 521 | Congenital heart disease | No | Alive | Negative | Negative | Negative | Negative | Negative |  |
| 522 | G6PD deficiency | No | Alive | Positive | Negative | Negative | 1:80 | Positive | Yes |
| 523 | None | No | Alive | Negative | Negative | Negative | Negative | Negative |  |
| 524 | None | Yes | Alive | Negative | Positive | Positive | Negative | Positive | Yes |
| 525 | None | No | Alive | Positive | Negative | Positive | 1:640 | Positive | Yes |
| 526 | Congenital heart disease | No | Alive | Negative | Negative | Negative | Negative | Negative |  |
| 527 | None | No | Alive | Negative | Negative | Negative | 1:40 | Negative |  |
| 528 | Bronchiolitis obliterans | No | Alive | Positive | Negative | Negative | 1:640 | Negative |  |
| 529 | None | No | Alive | Positive | Positive | Negative | 1:640 | Positive | Yes |
| 530 | None | No | Alive | Negative | Negative | Negative | Negative | Negative |  |
| 531 | None | No | Alive | Negative | Negative | Negative | Negative | Negative |  |
| 532 | Congenital heart disease | No | Alive | Positive | Negative | Negative | 1:80 | Negative |  |
| 533 | None | No | Alive | Positive | Positive | Negative | 1:640 | Positive | Yes |
| 534 | None | No | Alive | Positive | Negative | Negative | 1:640 | Negative |  |
| 535 | Lung cyst | No | Alive | Negative | Negative | Negative | Negative | Negative |  |
| 536 | Congenital heart disease | No | Alive | Negative | Negative | Negative | 1:320 | Negative |  |
| 537 | None | No | Alive | Positive | Negative | Negative | 1:320 | Negative |  |
| 538 | None | No | Alive | Negative | Negative | Negative | Negative | Negative |  |
| 539 | Brain lesions | No | Alive | Negative | Negative | Negative | 1:80 | Negative |  |
| 540 | None | No | Alive | Positive | Negative | Positive | 1:640 | Positive | Yes |
| 541 | None | No | Alive | Negative | Negative | Negative | Negative | Negative |  |
| 542 | None | No | Alive | Positive | Negative | Negative | 1:640 | Positive | Yes |
| 543 | None | No | Alive | Negative | Negative | Positive | Negative | Positive | No |
| 544 | None | No | Alive | Negative | Negative | Negative | Negative | Negative |  |
| 545 | None | No | Alive | Negative | Negative | Negative | Negative | Negative |  |
| 546 | None | No | Alive | Negative | Negative | Negative | Negative | Negative |  |
| 547 | None | Yes | Alive | Negative | Negative | Negative | Negative | Negative |  |
| 548 | None | No | Alive | Positive | Negative | Negative | 1:640 | Negative |  |
| 549 | None | No | Alive | Negative | Negative | Negative | 1:80 | Negative |  |
| 550 | None | No | Alive | Negative | Negative | Negative | Negative | Negative |  |
| 551 | None | No | Alive | Negative | Negative | Positive | 1:320 | Positive | Yes |
| 552 | None | No | Alive | Negative | Negative | Negative | Negative | Negative |  |
| 553 | Congenital heart disease | Yes | Alive | Negative | Negative | Negative | Negative | Negative |  |
| 554 | Congenital heart disease | Yes | Death | Negative | Negative | Negative | 1:40 | Negative |  |
| 555 | None | No | Alive | Negative | Negative | Negative | 1:320 | Negative |  |
| 556 | None | No | Alive | Negative | Negative | Negative | Negative | Negative |  |
| 557 | None | No | Alive | Positive | Negative | Negative | 1:640 | Negative |  |
| 558 | Congenital heart disease | No | Alive | Negative | Negative | Negative | Negative | Negative |  |
| 559 | Congenital heart disease | Yes | Alive | Negative | Negative | Negative | Negative | Positive | Yes |
| 560 | Congenital heart disease | No | Alive | Negative | Negative | Negative | Negative | Negative |  |
| 561 | None | No | Alive | Negative | Negative | Negative | Negative | Negative |  |
| 562 | None | No | Alive | Negative | Positive | Negative | Negative | Negative |  |
| 563 | None | No | Alive | Negative | Positive | Negative | 1:80 | Negative |  |
| 564 | None | No | Alive | Negative | Negative | Negative | Negative | Negative |  |
| 565 | None | No | Alive | Negative | Positive | Positive | 1:320 | Positive | Yes |
| 566 | Congenital heart disease | No | Alive | Negative | Positive | Negative | Negative | Negative |  |
| 567 | None | No | Alive | Negative | Negative | Negative | 1:640 | Positive | Yes |
| 568 | None | Yes | Alive | Negative | Negative | Negative | Negative | Negative |  |
| 569 | None | No | Alive | Negative | Negative | Negative | Negative | Negative |  |
| 570 | None | No | Alive | Negative | Negative | Negative | Negative | Negative |  |
| 571 | None | No | Alive | Negative | Negative | Negative | Negative | Negative |  |
| 572 | Congenital heart disease | Yes | Alive | Negative | Positive | Negative | Negative | Negative |  |
| 573 | None | No | Alive | Positive | Negative | Negative | 1:640 | Negative |  |
| 574 | Congenital heart disease | No | Alive | Negative | Positive | Negative | Negative | Negative |  |
| 575 | None | No | Alive | Positive | Positive | Negative | 1:640 | Negative |  |
| 576 | None | No | Alive | Negative | Negative | Negative | 1:40 | Negative |  |
| 577 | Congenital heart disease | No | Alive | Negative | Positive | Negative | 1:40 | Negative |  |
| 578 | None | No | Alive | Negative | Negative | Negative | 1:320 | Positive | No |
| 579 | None | No | Alive | Negative | Negative | Negative | Negative | Negative |  |
| 580 | None | No | Alive | Negative | Positive | Negative | 1:80 | Negative |  |
| 581 | None | No | Alive | Negative | Negative | Negative | 1:80 | Negative |  |
| 582 | Rhabdomyosarcoma | No | Alive | Negative | Negative | Negative | Negative | Negative |  |
| 583 | Congenital heart disease | No | Alive | Negative | Negative | Negative | 1:160 | Negative |  |
| 584 | None | No | Alive | Positive | Negative | Negative | 1:640 | Positive | Yes |
| 585 | None | No | Alive | Positive | Negative | Positive | 1:640 | Positive | Yes |
| 586 | Congenital heart disease | No | Alive | Negative | Positive | Negative | Negative | Negative |  |
| 587 | None | No | Alive | Negative | Negative | Negative | 1:320 | Positive | Yes |
| 588 | None | No | Alive | Positive | Positive | Negative | 1:640 | Positive | Yes |
| 589 | None | No | Alive | Negative | Positive | Negative | Negative | Negative |  |
| 590 | Tracheal stenosis | No | Alive | Negative | Negative | Negative | Negative | Negative |  |
| 591 | None | No | Alive | Positive | Negative | Negative | 1:640 | Negative |  |
| 592 | None | No | Alive | Positive | Negative | Positive | 1:640 | Positive | Yes |
| 593 | None | No | Alive | Positive | Negative | Negative | 1:640 | Negative |  |
| 594 | None | No | Alive | Positive | Negative | Negative | 1:640 | Negative |  |
| 595 | Congenital heart disease | No | Alive | Negative | Negative | Negative | Negative | Negative |  |
| 596 | None | No | Alive | Positive | Positive | Negative | 1:640 | Negative |  |
| 597 | None | No | Alive | Negative | Positive | Negative | Negative | Negative |  |
| 598 | None | No | Alive | Negative | Positive | Positive | 1:640 | Positive | Yes |
| 599 | None | No | Alive | Negative | Negative | Negative | 1:640 | Negative |  |
| 600 | None | No | Alive | Negative | Negative | Negative | Negative | Negative |  |
| 601 | None | No | Alive | Negative | Positive | Negative | 1:320 | Positive | Yes |
| 602 | None | No | Alive | Negative | Negative | Negative | 1:80 | Negative |  |
| 603 | None | No | Alive | Negative | Positive | Negative | 1:40 | Negative |  |
| 604 | None | No | Alive | Negative | Negative | Negative | Negative | Negative |  |
| 605 | None | No | Alive | Negative | Negative | Negative | 1:80 | Positive | Yes |
| 606 | None | No | Alive | Positive | Positive | Negative | 1:640 | Positive | Yes |
| 607 | None | No | Alive | Positive | Negative | Negative | 1:640 | Positive | Yes |
| 608 | None | No | Alive | Positive | Negative | Negative | 1:640 | Negative |  |
| 609 | Congenital heart disease | Yes | Alive | Negative | Negative | Negative | Negative | Negative |  |
| 610 | None | No | Alive | Negative | Negative | Positive | 1:640 | Positive | No |
| 611 | None | No | Alive | Negative | Positive | Negative | Negative | Negative |  |
| 612 | None | No | Alive | Positive | Negative | Negative | 1:40 | Positive | Yes |
| 613 | Congenital heart disease | No | Alive | Negative | Negative | Negative | Negative | Negative |  |
| 614 | None | No | Alive | Negative | Negative | Negative | Negative | Negative |  |
| 615 | None | No | Alive | Negative | Negative | Negative | Negative | Negative |  |
| 616 | None | No | Alive | Negative | Negative | Negative | Negative | Negative |  |
| 617 | None | No | Alive | Negative | Negative | Negative | Negative | Negative |  |
| 618 | None | No | Alive | Positive | Negative | Positive | 1:80 | Positive | Yes |
| 619 | None | No | Alive | Negative | Negative | Positive | 1:80 | Positive | Yes |
| 620 | Congenital heart disease | No | Alive | Negative | Negative | Positive | 1:40 | Positive | No |
| 621 | None | No | Alive | Negative | Negative | Positive | 1:320 | Positive | No |
| 622 | None | No | Alive | Positive | Negative | Negative | Negative | Negative |  |
| 623 | None | No | Alive | Negative | Negative | Negative | Negative | Negative |  |
| 624 | None | No | Alive | Negative | Negative | Negative | 1:160 | Negative |  |
| 625 | Congenital heart disease | No | Alive | Negative | Negative | Negative | 1:40 | Negative |  |
| 626 | None | No | Alive | Negative | Negative | Positive | Negative | Negative |  |
| 627 | None | No | Alive | Positive | Negative | Negative | 1:640 | Negative |  |
| 628 | None | No | Alive | Negative | Negative | Negative | 1:40 | Negative |  |
| 629 | None | No | Alive | Negative | Negative | Negative | Negative | Negative |  |
| 630 | Congenital hypothyroidism | No | Alive | Negative | Positive | Negative | Negative | Negative |  |
| 631 | None | No | Alive | Negative | Negative | Positive | 1:640 | Negative |  |
| 632 | None | No | Alive | Negative | Negative | Positive | 1:640 | Negative |  |
| 633 | None | Yes | Alive | Negative | Negative | Negative | Negative | Negative |  |
| 634 | None | No | Alive | Negative | Negative | Negative | 1:640 | Positive | Yes |
| 635 | None | No | Alive | Negative | Negative | Negative | Negative | Negative |  |
| 636 | None | Yes | Alive | Negative | Negative | Positive | 1:640 | Positive | Yes |
| 637 | None | No | Alive | Negative | Negative | Negative | 1:40 | Negative |  |
| 638 | None | No | Alive | Negative | Negative | Positive | 1:640 | Positive | Yes |
| 639 | None | No | Alive | Negative | Negative | Positive | 1:640 | Positive | Yes |
| 640 | None | No | Alive | Negative | Positive | Negative | 1:40 | Negative |  |
| 641 | None | No | Alive | Negative | Negative | Negative | 1:640 | Negative |  |
| 642 | None | Yes | Alive | Positive | Positive | Positive | 1:640 | Positive | Yes |
| 643 | None | No | Alive | Negative | Negative | Negative | 1:40 | Negative |  |
| 644 | None | No | Alive | Negative | Negative | Negative | Negative | Negative |  |
| 645 | None | No | Alive | Negative | Negative | Negative | Negative | Positive | No |
| 646 | None | No | Alive | Negative | Positive | Negative | Negative | Negative |  |
| 647 | Primary ciliary dyskinesia | No | Alive | Negative | Positive | Negative | 1:160 | Negative |  |
| 648 | None | No | Alive | Negative | Positive | Negative | Negative | Negative |  |
| 649 | None | No | Alive | Negative | Positive | Negative | 1:320 | Positive | No |
| 650 | None | No | Alive | Negative | Negative | Negative | 1:40 | Negative |  |
| 651 | None | No | Alive | Negative | Negative | Negative | Negative | Negative |  |
| 652 | None | No | Alive | Negative | Negative | Negative | Negative | Positive | No |
| 653 | None | No | Alive | Positive | Negative | Positive | 1:640 | Positive | Yes |
| 654 | None | No | Alive | Positive | Negative | Negative | 1:640 | Positive | Yes |
| 655 | Congenital heart disease | No | Alive | Negative | Negative | Negative | Negative | Positive | No |
| 656 | None | No | Alive | Positive | Negative | Negative | 1:640 | Positive | Yes |
| 657 | None | No | Alive | Negative | Negative | Positive | 1:320 | Positive | Yes |
| 658 | Congenital heart disease | No | Alive | Negative | Positive | Positive | Negative | Positive | Yes |
| 659 | None | No | Alive | Negative | Negative | Negative | Negative | Negative |  |
| 660 | None | No | Alive | Negative | Negative | Negative | 1:640 | Positive | Yes |
| 661 | Congenital heart disease | Yes | Alive | Negative | Positive | Negative | Negative | Positive | No |
| 662 | None | No | Alive | Negative | Negative | Negative | 1:320 | Positive | Yes |
| 663 | None | No | Alive | Positive | Negative | Positive | 1:640 | Positive | Yes |
| 664 | Thalassemia | No | Alive | Negative | Negative | Positive | 1:160 | Positive | Yes |
| 665 | None | No | Alive | Positive | Negative | Negative | 1:640 | Positive | Yes |
| 666 | Congenital heart disease | No | Alive | Negative | Negative | Negative | 1:640 | Positive | Yes |
| 667 | None | No | Alive | Negative | Negative | Negative | 1:80 | Negative |  |
| 668 | None | No | Alive | Negative | Negative | Negative | 1:80 | Negative |  |
| 669 | Congenital heart disease | No | Alive | Negative | Negative | Negative | 1:40 | Negative |  |
| 670 | Congenital heart disease | No | Alive | Positive | Positive | Negative | 1:160 | Negative |  |
| 671 | None | No | Alive | Positive | Negative | Positive | 1:640 | Positive | Yes |
| 672 | Congenital heart disease | Yes | Alive | Negative | Negative | Negative | Negative | Negative |  |
| 673 | Esophageal atresia | No | Alive | Negative | Negative | Negative | Negative | Negative |  |
| 674 | None | No | Alive | Negative | Positive | Negative | Negative | Negative |  |
| 675 | None | No | Alive | Positive | Negative | Positive | 1:640 | Positive | Yes |
| 676 | None | No | Alive | Negative | Negative | Negative | 1:640 | Positive | Yes |
| 677 | Congenital heart disease | No | Alive | Negative | Negative | Negative | 1:80 | Negative |  |
| 678 | None | No | Alive | Positive | Negative | Negative | 1:640 | Positive | Yes |
| 679 | Angiolymphoma | No | Alive | Negative | Positive | Negative | 1:40 | Positive | No |
| 680 | None | No | Alive | Negative | Negative | Positive | 1:640 | Positive | Yes |
| 681 | None | No | Alive | Negative | Negative | Negative | 1:160 | Positive | No |
| 682 | None | No | Alive | Negative | Positive | Negative | Negative | Negative |  |
| 683 | Congenital biliary atresia | No | Alive | Negative | Negative | Negative | 1:160 | Negative |  |
| 684 | None | No | Alive | Negative | Negative | Positive | 1:160 | Positive | Yes |
| 685 | None | No | Alive | Negative | Positive | Negative | 1:640 | Positive | Yes |
| 686 | Congenital heart disease | No | Alive | Negative | Positive | Negative | Negative | Negative |  |
| 687 | None | No | Alive | Negative | Negative | Negative | Negative | Negative |  |
| 688 | None | No | Alive | Negative | Negative | Positive | 1:80 | Positive | Yes |
| 689 | None | No | Alive | Positive | Negative | Positive | 1:640 | Positive | No |
| 690 | None | No | Alive | Negative | Negative | Negative | 1:320 | Positive | No |
| 691 | None | No | Alive | Positive | Negative | Negative | 1:640 | Positive | Yes |
| 692 | None | No | Alive | Negative | Negative | Negative | Negative | Negative |  |
| 693 | None | No | Alive | Positive | Negative | Negative | 1:640 | Positive | No |
| 694 | Congenital heart disease | Yes | Alive | Negative | Positive | Negative | Negative | Negative |  |
| 695 | None | No | Alive | Negative | Negative | Negative | 1:640 | Positive | No |
| 696 | None | No | Alive | Negative | Negative | Negative | 1:80 | Negative |  |
| 697 | None | No | Alive | Negative | Negative | Negative | 1:320 | Positive | Yes |
| 698 | None | No | Alive | Negative | Negative | Negative | 1:640 | Negative |  |
| 699 | None | No | Alive | Positive | Negative | Negative | 1:640 | Negative |  |
| 700 | Spinal Muscular Atrophy | Yes | Death | Negative | Positive | Negative | 1:160 | Negative |  |
| 701 | None | No | Alive | Negative | Negative | Negative | Negative | Negative |  |
| 702 | None | No | Alive | Positive | Negative | Positive | 1:640 | Positive | Yes |
| 703 | Congenital heart disease | No | Alive | Negative | Negative | Negative | Negative | Negative |  |
| 704 | Congenital anal atresia | No | Alive | Negative | Negative | Negative | Negative | Negative |  |
| 705 | None | No | Alive | Negative | Negative | Negative | 1:640 | Positive | Yes |
| 706 | None | No | Alive | Negative | Negative | Negative | Negative | Negative |  |
| 707 | None | No | Alive | Negative | Positive | Negative | 1:640 | Positive | Yes |
| 708 | None | No | Alive | Negative | Negative | Negative | 1:640 | Positive | Yes |
| 709 | None | No | Alive | Negative | Negative | Negative | Negative | Positive | Yes |
| 710 | None | No | Alive | Negative | Negative | Negative | 1:160 | Negative |  |
| 711 | None | Yes | Alive | Positive | Negative | Positive | 1:640 | Positive | Yes |
| 712 | None | No | Alive | Negative | Negative | Positive | 1:640 | Positive | Yes |
| 713 | Congenital heart disease | No | Alive | Negative | Negative | Negative | 1:640 | Positive | Yes |
| 714 | None | No | Alive | Negative | Negative | Negative | 1:80 | Negative |  |
| 715 | None | No | Alive | Negative | Negative | Negative | 1:40 | Negative |  |
| 716 | Congenital heart disease | Yes | Alive | Negative | Negative | Positive | Negative | Positive | Yes |
| 717 | None | No | Alive | Negative | Negative | Negative | Negative | Negative |  |
| 718 | None | No | Alive | Positive | Negative | Positive | 1:640 | Positive | Yes |
| 719 | None | No | Alive | Positive | Negative | Positive | 1:640 | Positive | Yes |
| 720 | None | No | Alive | Positive | Positive | Negative | 1:640 | Positive | No |
| 721 | Congenital anal atresia | No | Alive | Negative | Negative | Negative | 1:40 | Positive | Yes |
| 722 | None | No | Alive | Negative | Negative | Negative | 1:80 | Negative |  |
| 723 | None | No | Alive | Negative | Negative | Negative | 1:80 | Positive | Yes |
| 724 | None | No | Alive | Negative | Negative | Negative | Negative | Positive | Yes |
| 725 | None | No | Alive | Positive | Negative | Positive | 1:640 | Positive | Yes |
| 726 | None | No | Alive | Positive | Negative | Positive | 1:640 | Positive | Yes |
| 727 | Congenital heart disease | Yes | Death | Negative | Negative | Negative | Negative | Positive | Yes |
| 728 | None | Yes | Alive | Negative | Negative | Negative | 1:80 | Negative |  |
| 729 | None | No | Alive | Negative | Negative | Negative | Negative | Negative |  |
| 730 | None | No | Alive | Negative | Negative | Negative | 1:40 | Negative |  |
| 731 | None | No | Alive | Negative | Negative | Negative | Negative | Negative |  |
| 732 | None | No | Alive | Negative | Negative | Negative | 1:40 | Negative |  |
| 733 | None | No | Alive | Positive | Negative | Negative | 1:640 | Positive | Yes |
| 734 | None | No | Alive | Negative | Positive | Negative | 1:80 | Negative |  |
| 735 | None | No | Alive | Negative | Negative | Negative | 1:40 | Positive | Yes |
| 736 | Congenital biliary atresia | No | Alive | Negative | Negative | Negative | Negative | Negative |  |
| 737 | None | No | Alive | Positive | Negative | Negative | 1:640 | Positive | Yes |
| 738 | None | No | Alive | Positive | Negative | Positive | 1:640 | Positive | No |
| 739 | None | No | Alive | Negative | Negative | Positive | 1:640 | Positive | Yes |
| 740 | Congenital heart disease | No | Alive | Negative | Negative | Negative | Negative | Negative |  |
| 741 | None | No | Alive | Negative | Negative | Positive | 1:640 | Positive | Yes |
| 742 | None | No | Alive | Negative | Positive | Positive | 1:160 | Positive | Yes |
| 743 | Bronchiolitis obliterans | No | Alive | Positive | Negative | Negative | 1:640 | Negative |  |
| 744 | None | No | Alive | Negative | Positive | Negative | Negative | Negative |  |
| 745 | None | No | Alive | Negative | Negative | Positive | 1:640 | Positive | Yes |
| 746 | None | No | Alive | Negative | Negative | Negative | Negative | Positive | Yes |
| 747 | None | No | Alive | Negative | Negative | Negative | 1:320 | Positive | No |
| 748 | None | No | Alive | Positive | Positive | Positive | 1:640 | Positive | Yes |
| 749 | None | No | Alive | Negative | Negative | Negative | 1:640 | Negative |  |
| 750 | None | Yes | Alive | Negative | Negative | Negative | 1:640 | Positive | Yes |
| 751 | None | No | Alive | Negative | Positive | Negative | 1:320 | Positive | No |
| 752 | Congenital heart disease | No | Alive | Negative | Negative | Negative | Negative | Negative |  |
| 753 | Congenital heart disease | No | Alive | Negative | Negative | Negative | Negative | Positive | Yes |
| 754 | None | No | Alive | Negative | Negative | Negative | Negative | Negative |  |
| 755 | Congenital heart disease | No | Alive | Negative | Negative | Negative | Negative | Negative |  |
| 756 | None | Yes | Alive | Positive | Negative | Negative | 1:640 | Positive | Yes |
| 757 | None | No | Alive | Negative | Negative | Negative | Negative | Negative |  |
| 758 | Congenital heart disease | No | Alive | Negative | Negative | Negative | Negative | Negative |  |
| 759 | None | No | Alive | Negative | Negative | Positive | Negative | Negative |  |
| 760 | None | No | Alive | Positive | Negative | Negative | 1:640 | Positive | Yes |
| 761 | None | No | Alive | Negative | Negative | Positive | 1:640 | Positive | Yes |
| 762 | None | No | Alive | Negative | Negative | Negative | Negative | Negative |  |
| 763 | None | Yes | Alive | Negative | Positive | Positive | 1:640 | Positive | Yes |
| 764 | Congenital heart disease | No | Alive | Negative | Negative | Negative | Negative | Negative |  |
| 765 | None | No | Alive | Negative | Negative | Negative | Negative | Negative |  |
| 766 | None | No | Alive | Negative | Positive | Positive | 1:640 | Positive | Yes |
| 767 | None | No | Alive | Positive | Positive | Positive | 1:640 | Positive | Yes |
| 768 | None | No | Alive | Negative | Positive | Positive | 1:160 | Positive | Yes |
| 769 | Congenital biliary atresia | No | Alive | Negative | Negative | Negative | 1:640 | Negative |  |
| 770 | None | No | Alive | Negative | Negative | Negative | Negative | Negative |  |
| 771 | None | No | Alive | Negative | Negative | Positive | 1:320 | Positive | Yes |
| 772 | None | No | Alive | Negative | Negative | Positive | Negative | Positive | Yes |
| 773 | None | No | Alive | Negative | Negative | Positive | Negative | Positive | Yes |
| 774 | None | No | Alive | Negative | Negative | Negative | Negative | Negative |  |
| 775 | None | No | Alive | Negative | Positive | Positive | 1:640 | Positive | Yes |
| 776 | None | No | Alive | Negative | Positive | Positive | 1:640 | Positive | Yes |
| 777 | None | No | Alive | Positive | Negative | Negative | 1:320 | Positive | Yes |
| 778 | None | No | Alive | Negative | Negative | Negative | 1:640 | Positive | Yes |
| 779 | None | No | Alive | Negative | Negative | Positive | 1:640 | Positive | Yes |
| 780 | None | No | Alive | Negative | Negative | Positive | 1:160 | Positive | Yes |
| 781 | None | No | Alive | Positive | Negative | Negative | 1:640 | Positive | Yes |
| 782 | None | No | Alive | Positive | Negative | Negative | 1:640 | Positive | Yes |
| 783 | None | No | Alive | Positive | Negative | Negative | 1:640 | Positive | Yes |
| 784 | Brain lesions | No | Alive | Negative | Negative | Negative | Negative | Positive | No |
| 785 | None | No | Alive | Negative | Positive | Positive | Negative | Positive | No |
| 786 | None | No | Alive | Positive | Negative | Negative | 1:640 | Positive | Yes |
| 787 | None | No | Alive | Negative | Negative | Negative | Negative | Negative |  |
| 788 | None | No | Alive | Negative | Negative | Negative | Negative | Negative |  |
| 789 | None | No | Alive | Negative | Positive | Negative | 1:160 | Positive | Yes |
| 790 | None | No | Alive | Positive | Positive | Positive | 1:640 | Positive | Yes |
| 791 | Brain lesions | Yes | Alive | Negative | Negative | Negative | Negative | Negative |  |
| 792 | None | No | Alive | Negative | Positive | Positive | 1:80 | Positive | Yes |
| 793 | None | No | Alive | Negative | Negative | Negative | 1:40 | Negative |  |
| 794 | None | No | Alive | Positive | Negative | Positive | 1:640 | Positive | Yes |
| 795 | Congenital heart disease | Yes | Alive | Negative | Negative | Negative | 1:80 | Negative |  |
| 796 | None | No | Alive | Negative | Negative | Negative | 1:640 | Positive | Yes |
| 797 | None | No | Alive | Positive | Negative | Positive | 1:640 | Positive | Yes |
| 798 | None | No | Alive | Negative | Negative | Negative | Negative | Negative |  |
| 799 | None | No | Alive | Negative | Negative | Negative | Negative | Negative |  |
| 800 | None | No | Alive | Negative | Positive | Positive | Negative | Positive | Yes |
| 801 | None | No | Alive | Negative | Positive | Positive | 1:160 | Positive | Yes |
| 802 | None | No | Alive | Positive | Negative | Positive | 1:640 | Positive | Yes |
| 803 | None | No | Alive | Negative | Negative | Negative | Negative | Negative |  |
| 804 | None | No | Alive | Positive | Negative | Negative | 1:160 | Negative |  |
| 805 | None | No | Alive | Positive | Negative | Negative | 1:80 | Positive | Yes |
| 806 | None | No | Alive | Positive | Negative | Negative | 1:640 | Positive | No |
| 807 | None | No | Alive | Negative | Negative | Positive | 1:80 | Positive | Yes |
| 808 | None | Yes | Alive | Negative | Negative | Negative | Negative | Negative |  |
| 809 | None | Yes | Alive | Negative | Positive | Positive | 1:80 | Positive | Yes |
| 810 | None | No | Alive | Negative | Negative | Negative | 1:320 | Negative |  |
| 811 | None | No | Alive | Negative | Negative | Positive | Negative | Positive | Yes |
| 812 | None | No | Alive | Negative | Negative | Positive | 1:640 | Positive | Yes |
| 813 | None | No | Alive | Negative | Positive | Positive | 1:640 | Positive | Yes |
| 814 | None | No | Alive | Negative | Negative | Negative | 1:40 | Negative |  |
| 815 | None | No | Alive | Negative | Negative | Negative | Negative | Negative |  |
| 816 | None | No | Alive | Positive | Positive | Positive | 1:640 | Positive | Yes |
| 817 | None | No | Alive | Negative | Positive | Positive | 1:80 | Positive | No |
| 818 | None | No | Alive | Positive | Negative | Negative | 1:640 | Positive | Yes |
| 819 | None | No | Alive | Negative | Positive | Positive | 1:80 | Positive | Yes |
| 820 | None | No | Alive | Positive | Positive | Positive | 1:640 | Positive | Yes |
| 821 | None | No | Alive | Negative | Positive | Positive | 1:640 | Positive | Yes |
| 822 | None | No | Alive | Negative | Positive | Positive | 1:640 | Positive | Yes |
| 823 | None | No | Alive | Positive | Negative | Negative | 1:640 | Positive | Yes |
| 824 | None | No | Alive | Negative | Positive | Positive | 1:640 | Positive | Yes |
| 825 | None | No | Alive | Negative | Positive | Positive | 1:160 | Positive | Yes |
| 826 | None | No | Alive | Negative | Positive | Negative | 1:640 | Negative |  |
| 827 | None | No | Alive | Negative | Negative | Positive | 1:640 | Positive | Yes |
| 828 | None | No | Alive | Positive | Negative | Positive | 1:640 | Positive | Yes |
| 829 | None | No | Alive | Positive | Positive | Positive | 1:640 | Positive | Yes |
| 830 | None | No | Alive | Negative | Negative | Negative | Negative | Negative |  |
